# Stroke prevention in atrial fibrillation and ‘real world’ adherence to guidelines in the Balkan Region: The BALKAN-AF Survey

**DOI:** 10.1038/srep20432

**Published:** 2016-02-12

**Authors:** Tatjana S. Potpara, Gheorghe-Andrei Dan, Elina Trendafilova, Artan Goda, Zumreta Kusljugic, Sime Manola, Ljilja Music, Rodica Musetescu, Elisabeta Badila, Gorana Mitic, Vilma Paparisto, Elena S. Dimitrova, Marija M. Polovina, Stanislav L. Petranov, Hortensia Djergo, Daniela Loncar, Amira Bijedic, Sandro Brusich, Gregory Y. H. Lip, The BALKAN-AF Investigators, Tatjana S. Potpara, Marija Polovina, Srdjan Milanov, Marija Pavlovic, Marijana Petrovic, Stefan Simovic, Gorana Mitic, Marko Milanov, Jelena Savic, Sanja Gnip, Pavica Radovic, Snezana Markovic, Ivana Koncarevic, Jelena Gavrilovic, Tijana Acimovic, Dijana Djikic, Semir Malic, Jusuf Hodzic, Milovan Stojanovic, Marina Deljanin Ilic, Milan Zlatar, Dragan Matic, Snezana Lazic, Vladan Peric, Sanja Markovic, Snezana Kovacevic, Aleksandra Arandjelovic, Milika Asanin, Marija Zdravkovic, Gheorghe-Andrei Dan, Anca Breha, Anca Rodica Dan, Rodica Musetescu, Mircea Ioachim Popescu, Elisabeta Badila, Catalina Arsenescu Georgescu, Sorina Pop, Raluca Popescu, Simina Neamtu, Floriana Oancea, Elina Trendafilova, Elena Dimitrova, Evgenii Goshev, Anna Velichkova, Stanislav Petranov, Delyana Kamenova, Penka Kamenova, Svetoslava Elefterova, Valentin Shterev, Maria Zekova, Stela Diukiandzhieva, Boiko Dimitrov, Tihomir Sotirov, Valentina Simeonova, Dimitrina Drianovska, Liliya Ivanova Vasileva Boiadzhieva, Darina Buchukova, Artan Goda, Vilma Paparisto, Hortensia Gjergo, Alma Mijo, Ervina Shirka, Viktor Gjini, Uliks Ekmekciu, Ina Refatllari, Zumreta Kusljugic, Daniela Loncar, Denis Mrsic, Hazim Tulumovic, Belma Pojskic, Alma Sijamija, Amira Bijedic, Indira Karamujic, Irma Bijedic, Sanela Halilovic, Sekib Sokolovic, Sime Manola, Ivan Zeljkovic, Nikola Pavlovic, Vjekoslav Radeljic, Sandro Brusich, Ante Anic, Melita Jeric, Petar Pekic, Kresimir Milas, Ljilja Music, Nebojsa Bulatovic, Ana Nenezic, Dijana Asanovic

**Affiliations:** 1 School of Medicine, Belgrade University, Belgrade, Serbia; 2 Cardiology Clinic, Clinical Centre of Serbia, Belgrade, Serbia; 3 Medicine University “Carol Davila”, Colentina University Hospital, Bucharest, Romania; 4 National Heart Hospital, Sofia, Bulgaria; 5 Clinic of Cardiology, University Hospital centre Mother Theresa, Tirana, Albania; 6 Cardiology Department, Clinic for internal diseases, Tuzla, Bosnia & Herzegovina; 7 Clinical Hospital Centre Sestre Milosrdnice, Zagreb, Croatia; 8 University Clinical Centre Podgorica, Podgorica, Montenegro; 9 Cardiology Centre, County Emergency Hospital, Craiova, Romania; 10 Medicine University “Carol Davila”, Emergency Clinical Hospital, Internal Medicine Department, Bucharest, Romania; 11 Clinical Centre Vojvodina, Novi Sad, Serbia; 12 Multiprofile Hospital for Active Treatment, Bourgas, Bulgaria; 13 Clinical Hospital Centre Rijeka, Rijeka, Croatia; 14 University of Birmingham Centre for Cardiovascular Sciences, City Hospital, Birmingham B18 7QH, UK; 15 Cardiology Clinic, University Clinical Center of Serbia, School of Medicine, Belgrade University, Belgrade; 16 Cardiology Clinic, University Clinical Center of Kragujevac, Kragujevac; 17 Hematology Clinic, University Clinical Center of Vojvodina, University of Novi Sad, Medical Faculty, Novi Sad; 18 University Clinical Center Zvezdara, Cardiology Department, Belgrade; 19 Hematology Clinic, University Clinical Center of Vojvodina, Novi Sad; 20 General Hospital, Pirot; 21 University Clinical Center Bezanijska kosa, Cardiology Department, Belgrade; 22 General Hospital Gracanica, Gracanica; 23 Clinic for Cardiovascular Diseases, Institute Niska Banja, Niska Banja; 24 Emergency Center, Coronary Care Unit, University Clinical Center of Serbia, Belgrade; 25 Internal Medicine Clinic, University Clinical Center of Pristina, Pristina; 26 General Hospital Sabac, Sabac; 27 University Clinical Center Zvezdara, Cardiology Department, School of medicine, Belgrade University, Belgrade; 28 Emergency Center, Coronary Care Unit, Clinical Center of Serbia, School of medicine, Belgrade University, Belgrade; 29 University Clinical Center Bezanijska kosa, Cardiology Department, School of medicine, Belgrade University, Belgrade; 30 Medicine University “Carol Davila”, Colentina University Hospital, Bucharest; 31 Cardiology Centre – County Emergency Hospital, Craiova; 32 Cardiology department, Emergency Hospital, Oradea; 33 Emergency Clinical Hospital, Internal Medicine Department, Bucharest; 34 Insitute for Cardiovascular Diseases ’Prof Dr George I.M. Georgescu’, Iasi; 35 General Practice in Cluj-Napoca, Cluj-Napoca; 36 Colentina University Hospital, Cardiology Department, Bucharest; 37 General Practice in Timisoara, Timisoara; 38 Cardiology Department, County Emergency Hospital, Sibiu; 39 National Heart Hospital, Coronary Care Unit, Sofia; 40 Health Center, Bugras, Bugras; 41 Health Center Vidin, Vidin; 42 District Hospital/MHAT, Ruse; 43 University Hospital, Varna; 44 Health Center, Varna; 45 University Hospital, Pleven; 46 Health Center Gorna Oryahovitza, Gorna Oryahovitza; 47 Community Hospital, Montana; 48 Community Hospital, Haskovo; 49 Health Center, Sofia; 50 MBAL Dr Stefan Cherkezov AD/ Second Internal Department, Veliko Trnovo; 51 University of Medicine, Sofia; 52 Clinic of Cardiology, University Hospital Center Mother Theresa, Tirana; 53 Regional Hospital Fier; 54 Clinic of Internal Medicine, University Hospital Center Mother Theresa, Tirana; 55 Clinic of Internal Medicine, Cardiology Department, University Clinical Center Tuzla, Medical Faculty, Tuzla; 56 Clinic of Internal Medicine, Department of Intensive Care and Therapy, University Clinical Center Tuzla, Tuzla; 57 General Hospital, Zenica; 58 General Hospital, Travnik; 59 Clinic of Internal Medicine, Cardiology Department, University Clinical Center Tuzla, Tuzla; 60 Clinic of Internal Medicine, University Clinical Center Tuzla, Tuzla; 61 Clinic of Heart ad Rheumatologic Diseases, University Clinical Center Sarajevo, Sarajevo; 62 Clinical Center “Sestre Milosrdnice”, Zagreb; 63 Clinical Center Rijeka, Rijeka; 64 General Hospital, Zadar; 65 General Hospital Varazdin, Varazdin; 66 Clinical Hospital “Sveti Duh“, Zagreb; 67 General Hospital, Pula; 68 Cardiology Clinic, University Clinical Center of Montenegro, University of Podgorica, Medical Faculty, Podgorica; 69 Cardiology Clinic, University Clinical Center of Montenegro, Podgorica

## Abstract

Data on the management of atrial fibrillation (AF) in the Balkan Region are limited. The Serbian AF Association (SAFA) prospectively investigated contemporary ‘real-world’ AF management in clinical practice in Albania, Bosnia&Herzegovina, Bulgaria, Croatia, Montenegro, Romania and Serbia through a 14-week (December 2014-February 2015) prospective, multicentre survey of consecutive AF patients. We report the results pertinent to stroke prevention strategies. Of 2712 enrolled patients, 2663 (98.2%) with complete data were included in this analysis (mean age 69.1 ± 10.9 years, female 44.6%). Overall, 1960 patients (73.6%) received oral anticoagulants (OAC) and 762 (28.6%) received antiplatelet drugs. Of patients given OAC, 17.2% received non-vitamin K antagonist oral anticoagulants (NOACs). CHA_2_DS_2_-VASc score was not significantly associated with OAC use. Of the ‘truly low-risk’ patients (CHA_2_DS_2_-VASc = 0 [males], or 1 [females]) 56.5% received OAC. Time in Therapeutic Range (TTR) was available in only 18.7% of patients (mean TTR: 49.5% ± 22.3%). Age ≥ 80 years, prior myocardial infarction and paroxysmal AF were independent predictors of OAC non-use. Our survey shows a relatively high overall use of OAC in AF patients, but with low quality of vitamin K antagonist therapy and insufficient adherence to AF guidelines. Additional efforts are needed to improve AF-related thromboprophylaxis in clinical practice in the Balkan Region.

In parallel with increasing global burden of atrial fibrillation (AF), accumulating high-quality evidence from randomized clinical trials on AF management inform frequent updates of AF guidelines^1^. However, guideline implementation into daily clinical practice might be incomplete for many reasons and monitoring of routine practice through ongoing large, well-conducted long-term registries^2^,^3^ helps to understand and attenuate barriers for evidence-based management of AF in ‘real-world’ setting.

Recent reports from contemporary European AF registries have provided important insights into AF management in clinical practice^2^,^3^,^4^,^5^, including the observation of certain regional differences in management across Europe^6^. Most countries from the Balkan Region (comprising an area of >50 million inhabitants) were not participating in prior registries, and in contrast to other European regions, ‘real-world’ data on the management of AF in Balkan countries are limited.

In this study, we investigated contemporary real-world patterns of AF management in the Balkan Region through a prospective 14-week survey of consecutive AF patients in clinical practice, and we report the results pertinent to stroke prevention.

## Methods

### Study design and patient selection

A detailed report on the Balkan-AF study protocol has been published^7^. A 14-week prospective, multicentre ‘snapshot’ Balkan-AF survey of consecutive patients with electrocardiographically documented AF, who were seen by cardiologists or internal medicine specialists (in centres where a cardiologist was not available), was conducted from December 2014 to February 2015 in Albania, Bosnia & Herzegovina, Bulgaria, Croatia, Montenegro, Romania and Serbia (a total of ~40 million inhabitants). The survey was designed and conducted by the Serbian Atrial Fibrillation Association (SAFA), which is a non-profit multidisciplinary association of expert physicians involved in AF management and AF research.

The survey was announced to the National Cardiology Societies and relevant Working Groups or associations in Albania, Bosnia & Herzegovina, Bulgaria, Croatia, Former Yugoslav Republic Macedonia, Montenegro, Romania, Slovenia and Serbia. In the participating countries Balkan-AF survey was approved by the national and/or local Institutional Review Board, or the need for approval was waived according to the regulations in the respective country. In concordance with the local policy, a signed patient informed consent was obtained from each patient before enrolment. The study protocol conforms to the ethical guidelines of the 1975 Declaration of Helsinki as reflected in a priori approval by the institution’s human research committee.

Each country participated with university and non-university hospitals and outpatient health centres in- and outside the capital cities. Patients younger than 18 years and patients with prosthetic mechanical heart valves or significant valve disease requiring surgical repair were not included.

### Data collection

Data were collected via a web-based electronic case report form (CRF) with a range of pre-specified plausibility checks for the entries. The CRF was formulated to obtain the information on patients’ characteristics including demographics, cardiovascular risk factors, medical history, AF-related data regarding symptoms, prior history of AF, AF clinical type, prior use of antithrombotic medication, antiarrhythmic drugs or other therapies, health care setting (i.e., university/non-university health centre, in- or outside the capital city, in-hospital or outpatient, internal medicine specialist/cardiologist, main reason for current visit/hospitalization, emergency or non-emergency setting, length of hospitalization, etc.) and patient’s presentation, AF management at enrolling visit or hospitalization (i.e., medication, cardioversion, AF ablation) and further management strategy post discharge, and diagnostic procedures performed due to AF during enrolling visit/hospitalization or within the last 12 months (the latter was not applicable to patients with first-diagnosed AF). A detailed list of cardiovascular risk factors, diseases and risk scores definitions used in the Balkan-AF survey is provided in the Supplementary Appendix 1.

Systematic monitoring of centres was not performed due to the relatively short duration of the survey. The national coordinators and all investigators are the guarantors of the consecutiveness of enrolment, accuracy and completeness of data. The CRF, patient files, and medical records (paper or database) serve as source documents.

### Statistical analysis

Following a test of statistical normality, continuous variables were presented as mean with standard deviation (SD), or with a skewed distribution as median with interquartile range (IQR, 25^th^–75^th^ quartile). Categorical variables were reported as counts with percentages. The Student t-test was used for comparison of continuous variables with normal distribution, and Mann-Whitney test for continuous variables with skewed distribution. Differences in categorical variables were tested by Chi-square test.

Univariate and multivariable logistic regression analyses were used to investigate the associations of variables shown in Table 1 (that is, demographic data, patient clinical characteristics and AF characteristics) and health care setting with the use of oral anticoagulants (OAC) and other antithrombotic therapies (that is, antiplatelet drugs), as prescribed at discharge from enrolling visit or hospitalization. Variables statistically significant on univariate analysis were entered into the multivariable model to identify independent predictors of OAC use. All analyses were adjusted for country code, to account for differences in the health care systems among the participating countries.

Because the main reason for enrolling visit or hospitalization could have been either AF or some other condition, we have performed two sensitivity analyses. First, we excluded patients seen for other reasons (in whom the use of OAC might have been influenced by other condition) and performed the analysis of OAC use as in the main cohort. Second, we excluded patients presenting with an acute coronary syndrome and then performed the OAC use analyses in the rest of the main cohort.

All results are reported as Odds Ratio (OR) with 95% Confidence Interval (CI). All statistical analyses were performed using SPSS 20.0 software package (SPSS Inc., Chicago, Illinois). A two-sided P value of <0.05 was considered statistically significant.

## Results

A total of 2712 patients were enrolled in 49 centres from seven Balkan countries; 27 centres (55.1%) were university hospitals enrolling 2161 patients (86.6%). Eighteen centres (36.7%) were situated in the capital cities and enrolled 1241 patients (45.8%). A total of 2147 patients (79.2%) were enrolled by cardiologists, and 717 patients (26.8%) were seen in outpatient setting. Full data on antithrombotic therapy prescribed at current visit/hospitalization were available in 2663 patients (98.2%) and those patients were included in this analysis.

Demographic data (mean age 69.1 ± 10.9 years, range 18–96; female 44.6%), clinical characteristics of the study population and AF characteristics are shown in Table 1.

### Stroke and bleeding risk profile

**Figure 1:**
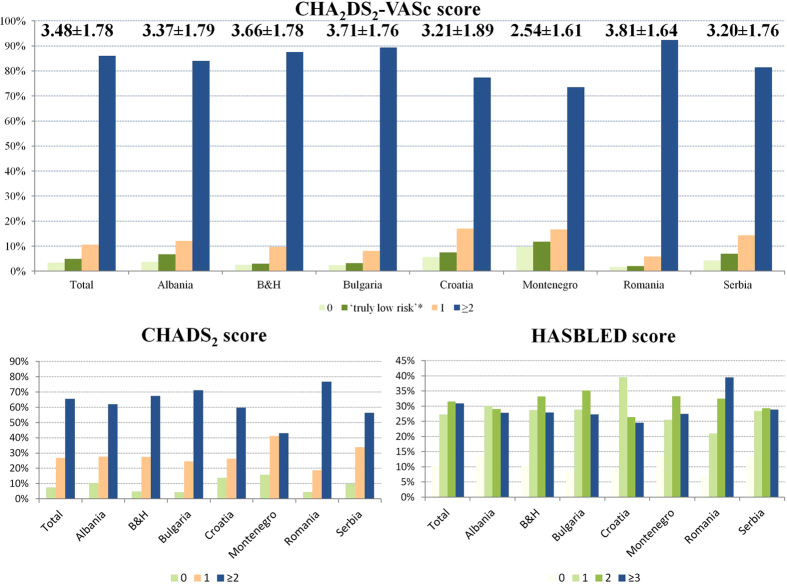
Stroke and bleeding risk. ‘Truly low-risk’: CHA_2_DS_2_-VASc = 0 in males, or CHA_2_DS_2_-VASc = 1 in females; B&H: Bosnia & Herzegovina.

Mean CHA_2_DS_2_-VASc score was 3.48 ± 1.77 (range 0–9, median 3.0, IQR 2.0–5.0), and a score of ≥2 was present in 2290 patients (86.0%). The mean CHADS_2_ score was 2.15 ± 1.29 (≥2 in 65.6% of patients), and mean HASBLED score was 1.97 ± 1.23 (range 0–6). Country-specific stroke and bleeding risk distribution is shown in Table 1, Fig. 1 and Supplemental Table 1.

### Antithrombotic therapies

Country-specific distribution of antithrombotic therapies is shown in Table 2. Overall, 264 patients (9.9%) were not given any antithrombotic therapy, 1960 patients (73.6%) were prescribed OAC, and 762 patients (28.6%) received an antiplatelet drug. OAC as only antithrombotic drug was given to 1637 patients (61.5%), whilst an antiplatelet drug only was given to 320 patients (12.0%, and in 91.2% of patients that was aspirin).

NOACs (i.e., dabigatran, rivaroxaban or apixaban) were given to 338 patients (12.7% of the whole study population or 17.2% of patients receiving OAC).

One patient previously underwent a left atrial appendage closure device implantation.

### Adherence to guidelines

**Figure 2:**
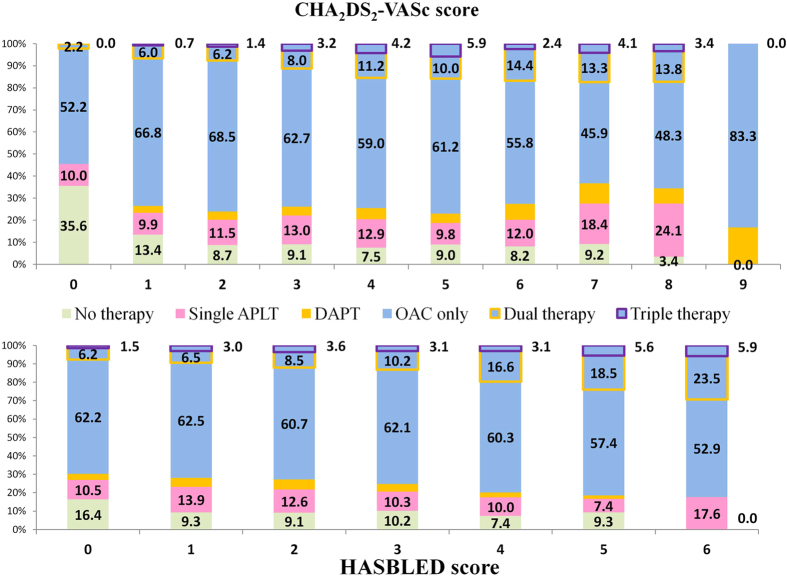
The use of antithrombotic therapies by CHA_2_DS_2_-VASc and HASBLED risk strata. APLT: antiplatelet therapy; DAPT: dual antiplatelet therapy; OAC: oral anticoagulant.

The proportions of OAC and other antithrombotic therapies by CHA_2_DS_2_-VASc and HASBLED score strata are shown in Fig. 2.

In the ‘truly’ low-risk group (that is, CHA_2_DS_2_-VASc score of 0 in males, or 1 in females) only 44 patients (33.6%) were not given any antithrombotic therapy, whilst 74 patients (56.5%) received OAC and 18 patients (13.7%) received an antiplatelet therapy, alone or in combination with OAC (3.8%). The use of OAC in ‘truly’ low-risk patients could be attributed to planned cardioversion or AF catheter ablation in only 21 patients (16.0%).

Of 2290 patients with CHA_2_DS_2_-VASc ≥2, 194 (8.5%) received no antithrombotic therapy, 1401 (61.2%) were given OAC only and 393 patients (17.2%) received an antiplatelet drug alone. A combination of OAC and an antiplatelet drug was given to 302 patients (13.2%).

There was no significant association between the CHA_2_DS_2_-VASc score and OAC prescription on multivariate analysis (Table 3). The non-relationship to CHADS_2_ score was similar (OR 1.06; 95% CI, 0.98–1.13, p = 0.119). The use of OAC increased with increasing HASBLED score, but the difference was significant only on univariate analysis (p = 0.042), Table 3.

The use of antiplatelet drugs was significantly associated with CHA_2_DS_2_-VASc score only on univariate analysis (Table 3).

### Determinants of OAC use

Independent predictors of the use of antithrombotic therapies are shown in Table 3, including significant univariate associations with the use of OAC or antiplatelet drug only (full univariate analyses list is shown in Supplemental Table 2).

### OAC monotherapy

On univariate analysis, most of the CHA_2_DS_2_-VASc score components were not significantly associated with OAC use, including prior stroke (OR 1.02; 0.76–1.37; p = 0.878). Patients with hypertension were more likely to use OAC, whilst older age (≥80 years) and coronary artery disease (CAD) were inversely associated with OAC use (Supplemental Table 2).

Increasing body mass index (BMI), mitral valve disease, dilated cardiomyopathy and thyroid disease were associated with increased use of OAC, whilst patients with chronic kidney disease (CKD) on dialysis and patients with chronic obstructive pulmonary disease (COPD) were less likely to use OAC (Supplemental Table 2).

**Figure 3:**
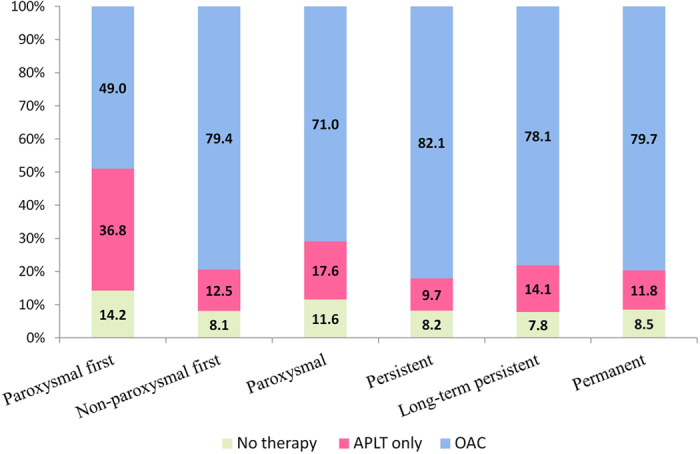
The use of oral anticoagulation by AF clinical type. APLT: antiplatelet therapy; OAC: oral anticoagulant.

The proportions of OAC use according to AF clinical type are shown in Fig. 3. Known history of AF was associated with increased use of OAC, whilst patients with paroxysmal AF were less likely to receive OAC. Patients treated in the health centres situated in the capital city or in university centres and patients managed by a cardiologist were more likely to use OAC compared to other patients (Supplemental Table 2).

Independent predictors of OAC use were hypertension, mitral valve disease, dilated cardiomyopathy, known history of AF and treatment in the capital city health centres, whilst age ≥80 years, prior MI and paroxysmal AF were independent predictors of OAC non-use (Table 3).

### Antiplatelet drug (aspirin) monotherapy

Independent predictors of aspirin monotherapy were age ≥80 years, CAD, aortic valve disease, COPD and paroxysmal AF, whilst BMI, known history of AF and treatment in the capital city health centre or treatment by a cardiologist were negatively associated with aspirin use (Table 3). On univariate analysis, increasing CHA_2_DS_2_-VASc score and prior transient ischemic attack (TIA) were also associated with increased use of aspirin only (Table 3).

### Other antithrombotic therapies

Independent predictors of dual antiplatelet drug therapy (DAPT) use were PCI, any CAD, COPD and paroxysmal AF, whilst patients with known history of AF were less likely to receive DAPT (Table 3). Independent predictors of the use of OAC combined with one or two antiplatelet drugs were CAD, PCI and treatment in a hospital-based centre, whilst patients aged ≥80 years were less likely to be given such therapy. Increasing HASBLED score was positively associated with the use of combined therapy (Table 3).

### Indices of VKA anticoagulation quality

An International Normalized Ratio (INR) obtained within previous 3 weeks was available in 946 (79.0%) of 1198 patients who were previously taking a VKA for at least 6 months or longer. The most recent INR value ranged from 1 to 10 (mean 2.42, SD 1.0, median 2.28), and was within the target range of 2.0 to 3.0 in 522 patients (55.2%) whilst in 281 (29.5%) and 143 patients (15.1%) the INR was below and above the target range, respectively.

The Time in Therapeutic Range (TTR) from the previous 3 months was available in only 224 patients (18.7%). Mean TTR was 49.5% ± 22.3% (median 50.0%, range 10–100%) and only 66 patients (29.5%) had a TTR of ≥65%. Labile INR was reported in 439 patients (36.6%) in whom TTR was not available.

### Sensitivity analyses

AF was the main reason for enrolling visit or hospitalization in 1329 patients (49.9%). Compared to the main cohort, these patients were younger (mean age 66.9 ± 11.3 years), with lower CHA_2_DS_2_-VASc (mean 2.95 ± 1.74) and lower HASBLED score (1.72 ± 1.19), all p < 0.01. OAC was given to 1000 patients (75.2%). Univariate and multivariable determinants of OAC use in patients with AF as the main reason for enrolling visit or hospitalization are shown in Supplemental Table 4. Similar to the main cohort, there was no significant relationship between the CHA_2_DS_2_-VASc score and the use of OAC in the multivariable analysis. The use of OAC was driven by the presence of hypertension, younger age (<75 years), increasing BMI, non-cardiac comorbidities (thyroid disease), centre localization (capital city) and university centre type, whilst patients with COPD, malignancy and paroxysmal AF were less likely to be prescribed OAC (Supplemental Table 4).

An acute coronary syndrome was the main reason for enrolling hospitalization in 206 patients (7.7%) and they were excluded from this analysis. In the remaining cohort of 2457 patients (mean age 68.9 ± 11.0 years, mean CHA_2_DS_2_-VASc 3.42 ± 1.77, mean HASBLED 1.97 ± 1.23) OAC was prescribed to 1843 patients (75.0%), and the use of OAC was driven by broadly similar determinants as in the main cohort (Supplemental Table 5).

## Discussion

This snapshot survey provides, for the first time, a contemporary insight into routine clinical practice in AF management from a large region of Europe where data on AF management are generally scarce, especially since countries participating in this survey were largely under-represented in recent European AF surveys^2^,^4^,^5^. This survey therefore complements the European AF data and reduces a gap in the European ‘map’ of contemporary real-world management of AF.

Our findings suggest that the overall use of OAC for stroke prevention in the Balkan region is relatively high (~74%), but poorly associated with individual patient stroke risk as recommended by the European Society of Cardiology guidelines^1^. Whilst mostly VKA were used, the quality of anticoagulation was poor, with less than a third of patients having a TTR of ≥65%. Overall, the use of NOACs was slightly higher than recently reported in a ‘real-world’ European survey^3^, and the use of antiplatelet drugs was comparably high as in other parts of Europe. We also observed significant differences in the use of antithrombotic therapies according to the physician specialty and health centre location. Our results have important practical implications and may help in recognizing the ‘action points’ needed to improve the management of AF patients at risk of stroke in daily clinical practice in the Balkan region.

The Balkan-AF cohort stroke risk profile was broadly similar to recent data from the EURObservational Pilot AF Registry^3^, with some minor differences (e.g., hypertension, diabetes mellitus and prior stroke were slightly more prevalent, whilst CAD, HF and valvular disease were slightly less frequent in the Balkan-AF cohort). However, bleeding risk was higher, and the difference might possibly be driven by labile INRs in many patients.

The overall use of OAC in Balkan-AF cohort was close to that in recent European reports^3^,^4^, but was not significantly associated with CHA_2_DS_2_-VASc score, even when categorised to <2 vs. ≥2. Despite clear evidence of low stroke risk in male AF patients with a CHA_2_DS_2_-VASc score of 0 and those with a score of 1 due to female sex^8^,^9^,^10^,^11^,^12^, in whom no antithrombotic therapy is recommended^1^, as many as 56.5% of such patients in the Balkan-AF cohort received OAC despite only a minority being scheduled for cardioversion or AF ablation.

Some deviations from evidence-based stroke prevention strategies were evident in the Balkan-AF cohort. For example, elderly patients were more likely to receive aspirin, despite clear evidence of net benefit with OAC in elderly^13^,^14^. Also, patients with stable CAD were more likely to receive aspirin or DAPT, or OAC plus antiplatelet drugs instead of OAC monotherapy, although evidence showed that combining OAC with antiplatelet drugs in AF patients with stable vascular disease resulted only in increased risk of major bleeding with no additional reduction of thromboembolism^15^,^16^. Such patterns of OAC use were also noted in other European countries^6^.

Independent predictors of increased OAC use in the Balkan-AF survey were hypertension, dilated cardiomyopathy and mild-to-moderate mitral valve disease (essentially mitral regurgitation). In contrast to mitral stenosis, data on the risk of stroke in AF patients with mitral regurgitation are controversial^17^ and decisions on OAC should be driven by the presence of well documented stroke risk factors.

The ‘chronicity’ of AF strongly influenced OAC use in the Balkan-AF cohort, with a 56% lower probability for OAC in paroxysmal AF and a 49% greater probability of OAC therapy in patients with history of AF. A large body of evidence suggests that stroke risk is comparable with paroxysmal or permanent AF^18^,^19^. Recently, this has been challenged by a meta-analysis of two non-anticoagulated AF cohorts with systematically adjudicated adverse events which showed AF type to be a strong independent predictor of stroke^20^, but the annual stroke rate with paroxysmal AF was still sufficiently high (2.1%) to warrant OAC therapy^1^. Hence, the decision on OAC should be guided by the presence of conventional stroke risk factors.

The use of aspirin alone was high across all CHA_2_DS_2_-VASc score strata and increased from 10% to 17.2% with increasing score. Despite sufficient evidence of only modest efficacy and similar safety compared to OAC^13^,^14^, aspirin is still used as monotherapy in around 15% of AF patients in Europe^2^. Independent predictors of aspirin use in the Balkan-AF survey (i.e., advanced age, paroxysmal AF, COPD, mild-to-moderate aortic valve disease) likely reflect a mixture of misperceptions of aspirin better safety (e.g., in elderly) or of lower stroke risk (e.g., paroxysmal AF, COPD, etc.) and an unjustified favouring of aspirin over OAC (e.g., in patients with stable CAD or aortic valve disease).

However, the HASBLED score was not a significant determinant of OAC use in our cohort (indeed, the use of OAC increased with increasing HASBLED), suggesting that either the role of HASBLED was correctly interpreted (that is, the score was used to flag up modifiable bleeding risk factors, and not to preclude OAC use) or perhaps the score was ignored. The latter seems to be the case particularly with the use of combined OAC plus antiplatelet drug therapy, which increased with increasing HASBLED score. Alternatively, sicker patients might have needed such therapy more often.

Around 80% of AF patients in our survey were managed by a cardiologist, and these patients were less likely to receive aspirin. Patients managed in health centres in the capital cities less often received aspirin and more often were given OAC, the latter likely resulting from the clustering of tertiary health centres in the capital cities in most of participating countries. Of note, similar influence of clinical background and specialty of treating physicians on antithrombotic treatment strategies in AF patients has been also described in other European countries^21^. However, the availability of a cardiologist with proper level of expertise for stroke risk management in AF patients in real-world clinical practice may significantly differ among countries.

The signal of poor quality of anticoagulation with VKA in the Balkan-AF cohort (as reflected by a low proportion of patients with a TTR of ≥65% and high proportion of patients with labile INRs) is particularly worrisome and calls for urgent action, as suboptimal TTR (<65–70%) is associated with an excess of both stroke and bleeding (and mortality)^15^,^22^,^23^. Indeed, a TTR of >70% is recommended in guidelines and position documents, when VKAs are used^15^. Also, missing TTR in >80% of patients in the Balkan-AF cohort might indicate that the calculation of TTR is not commonly used in routine clinical practice in Balkan countries. Given the low quality of therapy with VKA compared to other European countries^24^, increasing use of NOACs (17% of all patients taking OAC) is encouraging, as it could facilitate adequate stroke prevention in Balkan countries. An analysis of factors influencing the choice of NOAC over VKA in Balkan countries is underway.

This study is limited by its observational snapshot registry design, but we made every effort to include consecutive patients. Although we tried to capture a sample representative of real-world clinical practice by recruiting a range of different types of centres in each country (i.e., university and non-university hospitals and outpatient centres in- and outside the capital cities), there still may be a selection bias due to variable health care setting in the participating countries.

The proportion of cardiologists versus internal medicine specialists participating in the Balkan-AF survey may not fully reflect daily practice in the participating countries, since we might have not adequately covered the rural areas. Still, participating centres situated outside capital cities enrolled about 55% of patients, and in smaller countries many AF patients are often referred to the tertiary centres at least for initial evaluation.

Since TTR was available in a small proportion of patients treated with OAC, our results may under- or overestimate the quality of VKA treatment in our cohort, and we cannot conclude whether or not VKA therapy is systematically monitored in clinical practice.

## Conclusion

This survey provide important insights into contemporary routine practices for stroke prevention in AF patients in Balkan countries, thus reducing a gap in the European ‘map’ of contemporary real world management of AF. Our results show a broadly similar patient stroke risk profile in the Balkan Region and similar OAC use as in other European countries. However, routine practices for stroke prevention in AF patients in Balkan countries are less influenced by the presence of conventional well documented stroke risk factors in AF patients, and the quality of VKA therapy is low. Thus, additional efforts are needed to increase the adherence to AF guidelines and improve the management of AF-related risk of stroke in routine clinical practice in the Balkan Region.

## Additional Information

**How to cite this article**: Potpara, T. S. *et al*. Stroke prevention in atrial fibrillation and ‘real world’ adherence to guidelines in the Balkan Region: The BALKAN-AF Survey. *Sci. Rep*. **6**, 20432; doi: 10.1038/srep20432 (2016).

## Supplementary Material

Supplementary Information

## Figures and Tables

**Table 1:** Patient demographic data, clinical characteristics and AF characteristics.

	Total	Albania	B&H	Bulgaria	Croatia	Montenegro	Romania	Serbia
	*n *=* 2663*	*n *=* 313*	*n *=* 265*	*n *=* 443*	*n *=* 159*	*n *=* 102*	*n *=* 699*	*n *=* 682*
*Demographic*								
Age, years (mean ± SD)	69.1 ± 10.9	68.2 ± 10.2	69.2 ± 10.6	70.2 ± 10.7	69.6 ± 11.1	65.0 ± 10.8	70.9 ± 10.8	67.4 ± 11.0
Age ≥ 65–74 years (%)	878 (33.0)	114 (36.4)	99 (37.4)	139 (31.4)	46 (28.9)	39 (38.2)	215 (30.8)	226 (33.1)
Age ≥ 75 years (%)	942 (35.4)	94 (30.0)	87 (32.8)	176 (39.7)	62 (39.0)	20 (19.6)	301 (43.1)	202 (29.6)
Age ≥ 80 years (%)	418 (17.7)	39 (12.5)	40 (15.1)	76 (17.2)	31 (19.5)	6 (5.9)	143 (20.5)	83 (12.2)
Female sex (%)	1188 (44.6)	151 (48.2)	115 (43.4)	189 (42.7)	67 (42.1)	35 (34.3)	325 (46.5)	306 (44.9)
Cigarette smoking ever (%)	776 (29.1)	111 (35.5)	66 (24.9)	102 (23.0)	48 (30.2)	38 (37.3)	154 (22.0)	257 (37.7)
Cigarette smoking current (%)	339 (12.7)	74 (23.6)	32 (12.1)	48 (10.8)	29 (18.2)	22 (21.6)	58 (8.3)	76 (11.1)
Alcohol abuse (%)	110 (4.1)	30 (9.6)	9 (3.4)	35 (7.9)	4 (2.5)	0 (0.0)	20 (2.9)	12 (1.8)
Body mass index (mean ± SD)	27.7 ± 4.4	28.0 ± 3.9	26.8 ± 4.3	27.9 ± 3.8	27.8 ± 3.4	27.3 ± 3.7	28.5 ± 5.1	27.2 ± 4.3
Systolic BP, mmHg (mean ± SD)	134.6 ± 22.0	131.5 ± 25.0	133.3 ± 24.6	135.2 ± 19.0	137.5 ± 19.3	137.7 ± 19.6	136.2 ± 24.0	133.4 ± 19.8
Diastolic BP, mmHg (mean ± SD)	81.0 ± 12.2	81.4 ± 13.7	81.5 ± 12.7	81.6 ± 11.1	83.6 ± 10.0	86.8 ± 10.7	78.8 ± 13.1	81.0 ± 11.4
*Characteristics of AF*								
First diagnosed AF (%)	626 (23.5)	107 (34.4)	92 (34.7)	109 (24.6)	21 (13.2)	22 (21.6)	146 (20.9)	129 (18.9)
*Known history of AF*								
Paroxysmal (%)	554 (27.2)	43 (21.0)	23 (13.3)	77 (23.1)	45 (32.6)	31 (38.8)	106 (19.2)	229 (41.4)
Persistent (%)	319 (15.7)	13 (6.3)	12 (6.9)	72 (21.6)	46 (33.3)	13 (16.2)	77 (13.9)	86 (15.6)
Long-standing persistent (%)	64 (3.1)	7 (3.4)	12 (6.9)	7 (2.1)	6 (4.3)	1 (1.2)	9 (1.6)	22 (4.0)
Permanent (%)	1081 (53.1)	142 (69.3)	126 (72.8)	178 (53.2)	41 (29.7)	34 (42.5)	360 (65.1)	200 (36.2)
*Clinical parameters*								
Arterial hypertension (%)	2108 (79.2)	218 (69.6)	210 (79.2)	398 (89.8)	127 (79.9)	75 (73.5)	518 (74.1)	562 (82.4)
Heart failure ever (%)	1157 (43.5)	137 (43.8)	129 (48.9)	230 (51.9)	52 (32.7)	5 (4.9)	465 (66.6)	163 (23.9)
Signs of heart failure at present (%)	1104 (41.5)	113 (36.1)	129 (48.9)	223 (50.3)	34 (21.4)	3 (2.9)	454 (65.0)	124 (18.9)
Coronary artery disease (%)	816 (30.7)	99 (31.6)	106 (40.0)	147 (33.2)	35 (22.0)	16 (15.7)	256 (36.7)	157 (23.1)
Prior PCI/stenting (%)	224 (8.4)	35 (11.2)	6 (2.3)	62 (14.0)	9 (5.7)	8 (7.8)	50 (7.2)	54 (7.9)
Prior CABG (%)	97 (3.6)	12 (3.8)	13 (4.9)	17 (3.8)	3 (1.9)	1 (1.0)	15 (2.1)	36 (5.3)
Prior myocardial infarction (%)	365 (13.7)	50 (16.0)	53 (20.0)	40 (9.0)	13 (8.2)	8 (7.8)	115 (16.6)	86 (12.6)
Stable coronary artery disease (%)	592 (22.0)	64 (20.4)	100 (37.7)	85 (19.2)	26 (16.4)	8 (7.8)	206 (29.5)	103 (15.1)
Valvular disease (%)	933 (35.0)	69 (22.0)	60 (22.6)	159 (35.9)	39 (24.5)	3 (2.9)	414 (59.2)	189 (27.7)
Mitral valve disease (%)	844 (31.7)	53 (16.9)	54 (20.4)	131 (29.6)	34 (21.4)	1 (1.0)	388 (55.5)	183 (26.8)
Mitral valve regurgitation (%)	818 (30.7)	53 (16.9)	51 (19.2)	127 (28.7)	33 (20.8)	1 (1.0)	381 (54.5)	172 (25.2)
Aortic valve disease (%)	299 (11.2)	28 (8.9)	23 (8.7)	66 (14.9)	8 (5.0)	2 (2.0)	131 (18.7)	41 (6.0)
Dilated cardiomyopathy (%)	216 (8.1)	21 (6.7)	14 (5.3)	8 (1.8)	18 (11.3)	1 (1.0)	100 (14.3)	54 (7.9)
Hyperthrophic cardiomyopathy (%)	52 (2.0)	6 (1.9)	12 (4.5)	2 (0.5)	4 (2.5)	0 (0.0)	20 (2.9)	8 (1.2)
Restrictive cardiomyopathy (%)	4 (0.2)	0 (0.0)	0 (0.0)	0 (0.0)	1 (0.6)	0 (0.0)	3 (0.4)	0 (0.0)
Congenital heart disease (%)	7 (0.3)	0 (0.0)	0 (0.0)	1 (0.2)	0 (0.0)	0 (0.0)	2 (0.3)	4 (0.6)
Other cardiac disease (%)	206 (7.7)	11 (3.5)	8 (3.0)	9 (2.0)	4 (2.5)	0 (0.0)	92 (13.2)	82 (12.0)
Peripheral arterial disease (%)	122 (4.6)	13 (4.2)	7 (2.7)	19 (4.3)	5 (3.1)	2 (2.0)	40 (5.7)	36 (5.3)
Diabetes mellitus (%)	666 (25.0)	99 (31.6)	76 (28.7)	109 (24.6)	30 (18.9)	19 (18.6)	178 (25.5)	155 (22.7)
Chronic kidney disease (%)	411 (15.5)	36 (11.5)	33 (12.5)	78 (17.6)	21 (13.3)	1 (1.0)	164 (23.5)	78 (11.5)
Chronic hepatic disease (%)	96 (3.6)	7 (2.2)	9 (3.4)	13 (2.9)	2 (1.3)	0 (0.0)	54 (7.7)	11 (1.6)
Prior stroke (%)	280 (10.5)	29 (9.3)	40 (15.1)	47 (10.6)	13 (8.2)	7 (6.9)	65 (9.3)	79 (11.6)
Prior TIA (%)	83 (3.1)	16 (5.1)	18 (6.8)	14 (3.2)	5 (3.1)	0 (0.0)	14 (2.0)	16 (2.3)
Prior bleeding (%)	135 (5.0)	17 (5.4)	18 (6.8)	19 (4.3)	3 (1.9)	0 (0.0)	40 (5.7)	35 (5.0)
CHA_2_DS_2_-VASc score	3.48 ± 1.78	3.37 ± 1.79	3.66 ± 1.78	3.71 ± 1.76	3.21 ± 1.89	2.54 ± 1.61	3.81 ± 1.64	3.20 ± 1.76
HASBLED score	1.97 ± 1.23	1.87 ± 1.28	1.91 ± 1.18	1.92 ± 1.12	1.77 ± 1.17	1.87 ± 1.22	2.25 ± 1.26	1.86 ± 1.23

B&H: Bosnia & Herzegovina; SD: standard deviation; BP: blood pressure; AF: atrial fibrillation; PCI: percutaneous coronary intervention; CABG: coronary artery bypass grafting; TIA: transient ischemic attack.

**Table 2:** Country-specific distribution of OAC and antiplatelet therapies.

	Total	Albania	B&H	Bulgaria	Croatia	Montenegro	Romania	Serbia
	*n *=* 2663*	*n *=* 313*	*n *=* 265*	*n *=* 443*	*n *=* 159*	*n *=* 102*	*n *=* 699*	*n *=* 682*
No antithrombotic therapy (%)	264 (9.9)	23 (7.3)	28 (10.6)	46 (10.4)	15 (9.4)	11 (10.8)	67 (9.6)	74 (10.9)
Oral anticoagulant therapy-overall (%)	1960 (73.6)	229 (73.2)	133 (50.2)	319 (72.0)	134 (84.3)	72 (70.6)	534 (76.4)	539 (79.0)
VKAs (%)	1662 (60.9)	205 (65.5)	111 (41.9)	191 (43.1)	110 (69.2)	68 (66.7)	482 (69.0)	455 (66.7)
NOACs (%)	338 (12.7)	24 (7.7)	22 (8.3)	128 (28.9)	24 (15.1)	4 (3.9)	52 (7.4)	84 (12.3)
Oral anticoagulant therapy alone (%)	1637 (61.5)	162 (51.8)	124 (46.8)	286 (64.6)	121 (76.1)	50 (49.0)	446 (63.8)	448 (65.7)
Antiplatelet therapy (%)	762 (28.6)	128 (40.9)	113 (42.6)	111 (25.1)	23 (14.5)	41 (40.2)	186 (26.6)	160 (23.5)
Single antiplatelet drug only (%)	320 (12.0)	39 (12.5)	88 (33.2)	54 (12.2)	9 (5.7)	14 (13.7)	61 (8.7)	55 (8.1)
DAPT only (%)	119 (4.5)	22 (7.0)	16 (6.0)	24 (5.4)	1 (0.6)	5 (4.9)	37 (5.3)	14 (2.1)
Dual therapy^a^	240 (9.0)	51 (16.3)	9 (3.4)	17 (3.8)	9 (5.7)	14 (13.7)	74 (10.6)	66 (9.7)
Triple therapy^b^	83 (3.1)	16 (5.1)	0 (0.0)	16 (3.6)	4 (2.5)	8 (7.8)	14 (2.0)	25 (3.7)

B&H: Bosnia & Herzegovina; OAC: oral anticoagulant; VKA: vitamin-K antagonist; NOAC: non-vitamin K antagonist; DAPT: dual antiplatelet therapy.

^a^Dual therapy: OAC plus single antiplatelet agent.

^b^Triple therapy: OAC plus dual antiplatelet therapy.

**Table 3:** Determinants of the use of antithrombotic therapies for stroke prevention in AF patients (see also Supplemental Table 2–5).

Antithrombotic therapy	Univariate analysis (significant variables only)			Multivariate analysis			Antithrombotic therapy	Multivariate analysis		
*OAC only*	OR	95%CI	P	OR	95%CI	P	*DAPT*	OR	95%CI	P
HASBLED (cont. variable)	1.08	1.01–1.16	0.042				PCI	4.47	2.69–7.43	<0.001
Age≥80 years	0.62	0.50–0.78	<0.001	0.54	0.37–0.79	0.002	Coronary artery disease (any)	9.67	5.81–16.10	<0.001
Hypertension	1.67	1.36–2.05	<0.001	1.85	1.30–2.63	0.001	COPD	1.95	1.14–3.33	0.015
Prior MI	0.47	0.36–0.61	<0.001	0.58	0.37–0.91	0.018	Known history of AF	0.45	0.28–0.72	0.001
Prior PCI	0.32	0.22–0.47	<0.001				Paroxysmal AF	2.31	1.45–3.69	<0.001
Coronary artery disease (any)	0.49	0.41–0.60	<0.001							
Stable coronary artery disease	0.55	0.46–0.67	<0.001							
Mitral valve disease	1.40	1.15–1.70	0.001	1.56	1.07–2.28	0.021				
Dilated cardiomyopathy	2.06	1.40–3.04	<0.001	1.72	1.10–2.68	0.018	*Dual or triple therapy*	OR	95%CI	P
CKD on dialysis	0.16	0.04–0.60	0.007				Age≥80 years	0.58	0.39–0.88	0.009
COPD	0.73	0.57–0.94	0.016				PCI	3.69	2.46–5.46	<0.001
Thyroid disease	1.54	1.13–2.11	0.007				Coronary artery disease (any)	2.78	1.94–4.00	<0.001
Known history of AF	2.53	2.07–3.08	<0.001	1.51	1.04–2.20	0.032	Hospital–based centre	2.99	1.51–5.91	0.002
Paroxysmal AF	0.38	0.32–0.46	<0.001	0.44	0.32–0.62	<0.001	HASBLED (cont. variable)	1.24	1.11–1.38	<0.001
Body mass index	1.06	1.04–1.08	<0.001	1.04	1.01–1.08	0.031				
Centre in the capital city	2.02	1.68–2.43	<0.001	2.14	1.50–3.05	<0.001				
University centre	2.09	1.62–2.69	<0.001							
Cardiologist	1.55	1.26–1.92	<0.001							
*Antiplatelet drug only*	OR	95%CI	P	OR	95%CI	P	OAC (alone or in combination)	OR	95%CI	P
CHA_2_DS_2_-VASc (cont. variable)	1.08	1.01–1.16	0.021				HASBLED≥3	1.28	1.02–1.61	0.036
Age ≥80 years	1.88	1.42–2.49	<0.001	1.99	1.46–2.73	<0.001	Hypertension	1.76	1.40–2.22	<0.001
Coronary artery disease (any)	1.63	1.26–2.10	<0.001	1.35	1.03–1.77	0.033	Age≥80 years	0.52	0.39–0.67	<0.001
Stable coronary artery disease	1.59	1.23–2.07	<0.001				Coronary artery disease (any)	0.74	0.60–0.92	0.007
Aortic valve disease	2.52	1.64–3.86	<0.001	1.52	1.06–2.17	0.022	Mitral valve disease	1.29	1.03–1.62	0.030
Other cardiac disease	0.50	0.28–0.90	0.021				Dilated cardiomyopathy	1.76	1.15–2.67	0.009
Prior TIA	1.78	1.01–3.13	0.047				Thyroid disease	1.57	1.10–2.25	0.013
COPD	1.56	1.13–2.16	0.007	1.55	1.09–2.19	0.014	Known history of AF	1.50	1.16–1.93	0.002
Body mass index	0.96	0.93–0.99	0.003	0.97	0.94–0.99	0.033	Paroxysmal AF	0.32	0.24–0.42	<0.001
Known history of AF	0.57	0.43–0.74	<0.001	0.68	0.51–0.91	0.010	Body mass index	1.06	1.03–1.08	<0.001
Paroxysmal AF	1.91	1.50–2.48	<0.001	2.42	1.84–3.20	<0.001	Centre in the capital city	1.98	1.58–2.49	<0.001
Centre in the capital city	0.45	0.34–0.59	<0.001	0.40	0.30–0.54	<0.001	Treatment by a cardiologist	1.37	1.06–1.78	0.016
Treatment by a cardiologist	0.68	0.51–0.90	0.007	0.67	0.50–0.91	0.010				

OAC: oral anticoagulant; OR: Odds Ratio; CI: Confidence Interval; DAPT: dual antiplatelet drug therapy; AF: atrial fibrillation; MI: Myocardial infarction; PCI: percutaneous coronary intervention; CKD: chronic kidney disease; COPD: chronic obstructive pulmonary disease; TIA: transient ischemic attack.
