# Supplementary material for: Stroke prevention in atrial fibrillation and ‘real world’ adherence to guidelines in the Balkan Region: The BALKAN-AF Survey
Source: Sci Rep. 2016 Feb 12;6:20432. doi: 10.1038/srep20432 (PMC4751531; doi:10.1038/srep20432)
Supplement: Supplementary Information [file srep20432-s1.doc]

**Stroke prevention in atrial fibrillation and ‘real world’ adherence to guidelines in the Balkan Region: The BALKAN-AF Survey**

Tatjana S. Potpara^1,2^, Gheorghe-Andrei Dan^3^, Elina Trendafilova^4^, Artan Goda^5^, Zumreta Kusljugic^6^, Sime Manola^7^, Ljilja Music^8^, Rodica Musetescu^9^, Elisabeta Badila^10^, Gorana Mitic^11^, Vilma Paparisto^5^, Elena S. Dimitrova^4^, Marija M. Polovina^1,2^, Stanislav L. Petranov^12^, Hortensia Djergo^5^, Daniela Loncar^6^, Amira Bijedic^6^, Sandro Brusich^13^ and Gregory Y.H. Lip^1,14^, on behalf of the BALKAN-AF Investigators.

^1^School of Medicine, Belgrade University, Belgrade, Serbia,

^2^Cardiology Clinic, Clinical Centre of Serbia, Belgrade, Serbia,

^3^Medicine University "Carol Davila", Colentina University Hospital, Bucharest, Romania,

^4^ National Heart Hospital, Sofia, Bulgaria,

^5^Clinic of Cardiology, University Hospital centre Mother Theresa, Tirana, Albania,

^6^Cardiology Department, Clinic for internal diseases, Tuzla, Bosnia & Herzegovina,

^7^Clinical Hospital Centre Sestre Milosrdnice, Zagreb, Croatia,

^8^University Clinical Centre Podgorica, Podgorica, Montenegro,

^9^Cardiology Centre, County Emergency Hospital, Craiova, Romania,

^10^ Medicine University "Carol Davila", Emergency Clinical Hospital, Internal Medicine Department, Bucharest, Romania,

^11^Clinical Centre Vojvodina, Novi Sad, Serbia,

^12^Multiprofile Hospital for Active Treatment, Bourgas, Bulgaria,

^13^Clinical Hospital Centre Rijeka, Rijeka, Croatia,

^14^University of Birmingham Centre for Cardiovascular Sciences, City Hospital, Birmingham B18 7QH, UK.

**The BALKAN-AF Investigators:**

**SERBIA: Tatjana S. Potpara**, Cardiology Clinic, University Clinical Center of Serbia, School of Medicine, Belgrade University, Belgrade; **Marija Polovina**, Cardiology Clinic, University Clinical Center of Serbia, School of Medicine, Belgrade University, Belgrade; **Srdjan Milanov**, Cardiology Clinic, University Clinical Center of Kragujevac, Kragujevac; **Gorana Mitic**, Hematology Clinic, University Clinical Center of Vojvodina, University of Novi Sad, Medical Faculty, Novi Sad; **Marko Milanov**, University Clinical Center Zvezdara, Cardiology Department, Belgrade; **Jelena Savic**, Hematology Clinic, University Clinical Center of Vojvodina, Novi Sad; **Snezana Markovic**, General Hospital, Pirot; **Ivana Koncarevic**, University Clinical Center Bezanijska kosa, Cardiology Department, Belgrade; **Jelena Gavrilovic**, University Clinical Center Bezanijska kosa, Cardiology Department, Belgrade; **Marija Pavlovic**, Cardiology Clinic, University Clinical Center of Kragujevac, Kragujevac; **Dijana Djikic**, General Hospital Gracanica, Gracanica; **Marijana Petrovic**, Cardiology Clinic, University Clinical Center of Kragujevac, Kragujevac; **Stefan Simovic**, Cardiology Clinic, University Clinical Center of Kragujevac, Kragujevac; **Semir Malic**, General Hospital Gracanica, Gracanica; **Jusuf Hodzic**, General Hospital Gracanica, Gracanica; **Milovan Stojanovic**, Clinic for Cardiovascular Diseases, Institute Niska Banja, Niska Banja; **Sanja Gnip**, Hematology Clinic, University Clinical Center of Vojvodina, Novi Sad; **Milan Zlatar**, Emergency Center, Coronary Care Unit, University Clinical Center of Serbia, Belgrade; **Dragan Matic**, Emergency Center, Coronary Care Unit, University Clinical Center of Serbia, Belgrade; **Snezana Lazic**, Internal Medicine Clinic, University Clinical Center of Pristina, Pristina; **Tijana Acimovic**, University Clinical Center Bezanijska kosa, Cardiology Department, Belgrade; **Pavica Radovic**, Hematology Clinic, University Clinical Center of Vojvodina, Novi Sad; **Vladan Peric**, Internal Medicine Clinic, University Clinical Center of Pristina, Pristina; **Sanja Markovic**, Internal Medicine Clinic, University Clinical Center of Pristina, Pristina; **Snezana Kovacevic**, General Hospital Sabac, Sabac; **Aleksandra Arandjelovic**, University Clinical Center Zvezdara, Cardiology Department, School of medicine, Belgrade University, Belgrade; **Milika Asanin**, Emergency Center, Coronary Care Unit, Clinical Center of Serbia, School of medicine, Belgrade University, Belgrade; **Marija Zdravkovic**, University Clinical Center Bezanijska kosa, Cardiology Department, School of medicine, Belgrade University, Belgrade; **Marina Deljanin Ilic**, Clinic for Cardiovascular Diseases, Institute Niska Banja, Niska Banja; **ROMANIA: Gheorghe-Andrei Dan**, Medicine University "Carol Davila", Colentina University Hospital, Bucharest; **Rodica Musetescu**, Cardiology Centre – County Emergency Hospital, Craiova; **Mircea Ioachim Popescu**, Cardiology department, Emergency Hospital, Oradea; **Elisabeta Badila**, Emergency Clinical Hospital, Internal Medicine Department, Bucharest; **Catalina Arsenescu Georgescu**, Insitute for Cardiovascular Diseases ’Prof Dr George I.M. Georgescu’, Iasi; **Sorina Pop**, General Practice in Cluj-Napoca, Cluj-Napoca; **Raluca Popescu**, Colentina University Hospital, Cardiology Department, Bucharest; **Simina Neamtu**, General Practice in Timisoara, Timisoara; **Floriana Oancea**, Cardiology Department, County Emergency Hospital, Sibiu; **Anca Breha**, Medicine University "Carol Davila", Colentina University Hospital, Bucharest; **Anca Rodica Dan,** Medicine University "Carol Davila", Colentina University Hospital, Bucharest; **BULGARIA: Elina Trendafilova** , National Heart Hospital, Coronary Care Unit, Sofia; **Elena Dimitrova**, National Heart Hospital, Coronary Care Unit, Sofia; **Stanislav Petranov**, Health Center, Bugras, Bugras; **Delyana Kamenova**, Health Center Vidin, Vidin; **Penka Kamenova**, District Hospital/MHAT, Ruse; **Svetoslava Elefterova**, University Hospital, Varna; **Valentin Shterev**, Health Center, Varna; **Maria Zekova**, University Hospital, Pleven; **Stela Diukiandzhieva**, Health Center Gorna Oryahovitza, Gorna Oryahovitza; **Evgenii Goshev**, National Heart Hospital, Coronary Care Unit, Sofia; **Boiko Dimitrov**, Community Hospital, Montana; **Tihomir Sotirov**,Community Hospital, Haskovo; **Valentina Simeonova**, Health Center, Sofia; **Anna Velichkova**, National Heart Hospital, Coronary Care Unit, Sofia; **Dimitrina Drianovska**, Health Center, Sofia; **Liliya Ivanova Vasileva Boiadzhieva**, MBAL Dr Stefan Cherkezov AD/ Second Internal Department, Veliko Trnovo; **Darina Buchukova**, University of Medicine, Sofia; **ALBANIA: Artan Goda,** Clinic of Cardiology, University Hospital Center Mother Theresa, Tirana; **Vilma Paparisto**, Clinic of Cardiology, University Hospital Center Mother Theresa, Tirana; **Viktor Gjini**, Regional Hospital Fier; **Uliks Ekmekciu**, Clinic of Internal Medicine, University Hospital Center Mother Theresa, Tirana; **Hortensia Gjergo**, Clinic of Cardiology, University Hospital Center Mother Theresa, Tirana; **Alma Mijo**, Clinic of Cardiology, University Hospital Center Mother Theresa, Tirana; **Ervina Shirka**, Clinic of Cardiology, University Hospital Center Mother Theresa, Tirana; **Ina Refatllari**, Clinic of Internal Medicine, University Hospital Center Mother Theresa, Tirana; **BOSNIA & HERZEGOVINA: Zumreta Kusljugic**, Clinic of Internal Medicine, Cardiology Department, University Clinical Center Tuzla, Medical Faculty, Tuzla; **Daniela Loncar**, Clinic of Internal Medicine, Department of Intensive Care and Therapy, University Clinical Center Tuzla, Tuzla; **Belma Pojskic**, General Hospital, Zenica; **Denis Mrsic**, Clinic of Internal Medicine, Department of Intensive Care and Therapy, University Clinical Center Tuzla, Tuzla; **Alma Sijamija**, General Hospital, Travnik; **Amira Bijedic**, Clinic of Internal Medicine, Cardiology Department, University Clinical Center Tuzla, Tuzla; **Irma Bijedic**, Clinic of Internal Medicine, University Clinical Center Tuzla, Tuzla; **Indira Karamujic**, Clinic of Internal Medicine, Cardiology Department, University Clinical Center Tuzla, Tuzla; **Sanela Halilovic**, General Hospital Travnik; **Hazim Tulumovic**, Clinic of Internal Medicine, Department of Intensive Care and Therapy, University Clinical Center Tuzla, Tuzla; **Sekib Sokolovic**, Clinic of Heart ad Rheumatologic Diseases, University Clinical Center Sarajevo, Sarajevo; **CROATIA: Sime Manola**, Clinical Center „Sestre Milosrdnice“, Zagreb; **Sandro Brusich**, Clinical Center Rijeka, Rijeka; **Ivan Zeljkovic**, Clinical Center „Sestre Milosrdnice“, Zagreb; **Ante Anic**, General Hospital, Zadar; **Nikola Pavlovic**, Clinical Center „Sestre Milosrdnice“, Zagreb; **Vjekoslav Radeljic**, Clinical Center „Sestre Milosrdnice“, Zagreb; **Melita Jeric**, General Hospital Varazdin, Varazdin; **Petar Pekic**, Clinical Hospital „Sveti Duh“, Zagreb; **Kresimir Milas**, General Hospital, Pula; **MONTENEGRO: Ljilja Music**, Cardiology Clinic, University Clinical Center of Montenegro, University of Podgorica, Medical Faculty, Podgorica; **Ana Nenezic**, Cardiology Clinic, University Clinical Center of Montenegro, Podgorica; **Nebojsa Bulatovic**, Cardiology Clinic, University Clinical Center of Montenegro, University of Podgorica, Medical Faculty, Podgorica; **Dijana Asanovic**, Cardiology Clinic, University Clinical Center of Montenegro, Podgorica.

**Address for correspondence:**

Dr Tatjana Potpara, MD, PhD, FESC.

Cardiology Clinic, Clinical Center of Serbia, Visegradska 26, 11000 Belgrade, Serbia.

Tel: +381 11 3616319, Fax: +381 11 3616319; [tanjapotpara@gmail.com](mailto:tanjapotpara@gmail.com); [tatjana.potpara@mfub.bg.ac.rs](mailto:tatjana.potpara@mfub.bg.ac.rs)

**APPENDIX 1**

**Definitions used in the BALKAN-AF Survey**

**Acute coronary syndrome** – *i)* acute coronary syndrome with ST segment elevation - acute chest pain with persistent (>20 min) ST-segment elevation or (presumably) new left bundle branch block, *ii)* acute coronary syndrome without persistent ST segment elevation - acute chest pain with persistent or transient ST-segment depression or T-wave inversion, flat T waves, pseudo-normalization of T waves, or no ECG changes; if rise/fall in cardiac troponins was registered, acute coronary syndrome without persistent ST segment elevation was designated as non-ST elevation myocardial infarction, while normal troponin levels denoted unstable angina pectoris (1).

**Stable coronary artery disease** – episodes of reversible myocardial demand/supply mismatch, related to ischemia or hypoxia, which are usually inducible by exercise, emotion or other stress and reproducible — but, which may also be occurring spontaneously (2).

**Hypertension** – ≥140 mmHg systolic blood pressure and/or ≥90 mmHg diastolic blood pressure at office measurement (3).

**Heart failure** – symptoms (e.g. breathlessness, ankle swelling, fatigue) and signs (elevated jugular venous pressure, pulmonary crackles, and displaced apex beat) resulting from an abnormality of cardiac structure or function (4).

**New York Heart Association (NYHA) classification** - **Class I:** No limitation of physical activity. Ordinary physical activity does not cause undue breathlessness, fatigue, or palpitations. **Class II:** Slight limitation of physical activity. Comfortable at rest, but ordinary physical activity results in undue breathlessness, fatigue, or palpitations. **Class III:** Marked limitation of physical activity. Comfortable at rest, but less than ordinary physical activity results in undue breathlessness, fatigue, or palpitations. **Class IV:** Unable to carry on any physical activity without discomfort. Symptoms at rest can be present. If any physical activity is undertaken, discomfort is increased (4).

**Valvular heart disease –** intrinsic lesions affecting one or several components of the native heart valve apparatus including mild to moderate native heart valve stenosis, prolapse or regurgitation (5).

**Dilated cardiomyopathy –** ventricular enlargement and ventricular systolic dysfunction in the absence of significant coronary artery disease (6).

**Hypertrophic cardiomyopathy** - the presence of increased left ventricular wall thickness that is not solely explained by abnormal loading conditions (7).

**Restrictive cardiomyopathy –** impaired ventricular diastolic filling due to excessive rigidity of ventricular walls, without impairment of systolic function or evidence of pericardial disease (6).

**Congenital heart disease –** abnormality of cardiocirculatory structure and/or function that is present at birth, even if discovered later in life (8).

**AF clinical type** - *First diagnosed AF*: patients who presented with AF for the first time, irrespective of AF duration before diagnosis. *Paroxysmal AF*: self-terminating AF episodes, usually lasting 48 h - 7 days. *Persistent AF*: an AF episode that lasts longer than 7 days or requires termination by cardioversion, either with drugs or by direct current cardioversion*. Long-standing persistent AF:*  AF that has persisted for ≥1 year when it is decided to adopt a rhythm control strategy and attempt AF termination by cardioversion. *Permanent AF*: the presence of permanent AF is accepted by the patient and the physician and rhythm control interventions are not further pursued (9).

**EHRA symptom classification -** *EHRA I*: ‘No symptoms’; *EHRA II*: ‘Mild symptoms’ - normal daily activity not affected; *EHRA III*: ‘Severe symptoms’; normal daily activity affected; *EHRA IV*: ‘Disabling symptoms’; normal daily activity discontinued (9).

**CHADS_2_ score** – *C* - congestive heart failure; *H* – arterial hypertension; *A* - age ≥75 years; *D* – diabetes mellitus; *S_2_* – history of stroke or transient ischemic attack. Two points are assigned for a history of stroke or transient ischemic attack and 1 point each is assigned for age ≥ 75 years, a history of hypertension, diabetes, or heart failure (9).

**CHA_2_DS_2_-VASc** **score** - *C* - congestive heart failure or left ventricular systolic dysfunction (left ventricular ejection fraction ≤40%); *H* – arterial hypertension; *A_2_* - age ≥75 years; *D* – diabetes mellitus; *S_2_*– history of stroke or transient ischemic attack; *V* – vascular disease (prior myocardial infarction, peripheral artery disease, aortic plaque); *A* – age 65-74 years; *Sc* – sex category –female. Two points are assigned for a history of stroke or transient ischemic attack, or age ≥75; and 1 point each is assigned for age 65–74 years, hypertension, diabetes, heart failure/left ventricular systolic dysfunction, vascular disease and female sex (9).

**HASBLED score** – *H* – hypertension ( defined as systolic blood pressure ≥160 mmHg); *A* – Abnormal kidney function (presence of chronic dialysis or renal transplantation or serum creatinine ≥200 mmol/L. and or abnormal liver function (chronic hepatic disease such as cirrhosis or laboratory abnormalities denoting significant hepatic derangement such as bilirubin ≥2 x upper limit of normal, in association with aspartate aminotransferase/alanine aminotransferase/alkaline phosphatase ≥3 x upper limit normal); *B* – bleeding (previous bleeding events and/or predisposition to bleeding such as bleeding diathesis, anaemia, etc.); Labile International Normalized Ratios (INR) (unstable/high INRs or time in therapeutic range ≤60%); *D* - drugs/alcohol use (concomitant use of drugs, such as antiplatelet agents, non-steroidal anti-inflammatory drugs, or alcohol abuse). One point each is assigned for any of the HASBLED score components (9).

**Peripheral arterial disease –** atherothrombotic disease of the carotid, vertebral, upper extremity, mesenteric, renal, and lower extremity arterial vessels (10).

**Sleep apnea –** repetitive collapse of the upper airway during sleep with chronic intermittent hypoxia and recurrent arousals (11).

**COPD**  – persistent airflow limitation that is usually progressive and associated with an enhanced chronic inflammatory response in the airways (12).

**Anaemia –** Haemoglobin level in non-pregnant women <120 g/L and men <130 g/L (13).

**Diabetes mellitus –** fasting plasma glucose ≥ 7.0 mmol/L, and/or 2-h post-load plasma glucose ≥11.0 mmol/L, and/or HbA_1c_ ≥6.5% (14).

**Chronic kidney disease -** kidney damage, as evidenced by the presence of a structural abnormality or persistent hematuria and/or proteinuria, and/or reduced kidney function, as determined by a decreased glomerular filtration rate (eGFR <60 mL/min/1.73 m^2^) (15).

**Chronic liver disease** - patients presenting with signs and symptoms of chronic liver disease or has risk factors for chronic liver disease (e.g., alcohol abuse, risk of viral hepatitis, obesity) with confirmed laboratory abnormalities (e.g., elevated aspartate aminotransferase/alanine aminotransferase) or positive screen for serologic markers of liver disease (16).

**Thyroid disease -** *Hyperthyreosis* **:** TSH below the lower limit of normal and FT4 and FT3 above the upper limit of normal; *Hypothyreosis*: TSH above the upper limit of normal and FT4 and FT3 below the lower limit of normal

**Malignancy –**confirmed solid organ or hematological malignancy currently under treatment or previously treated and considered as cured.

**Stroke** - a focal neurologic deficit, from a nontraumatic cause, lasting at least 24 hours, categorized as ischemic (with or without hemorrhagic transformation), hemorrhagic, or of uncertain type (in the case of patients who did not undergo brain imaging or in whom an autopsy was not performed) (17).

**Transient ischemic attack** - a focal neurologic deficit, from a nontraumatic cause, lasting less than 24 hours (17).

**Peripheral embolism** – clinical history consistent with an acute loss of blood flow to a peripheral artery (or arteries) supported by evidence of embolism from surgical specimens, autopsy, angiography, vascular imaging, or other objective testing (17).

**Bleeding -** *ISTH definition of major bleeding*: *i)* Fatal bleeding, and/or *ii)* symptomatic bleeding in a critical area or organ, such as intracranial, intraspinal, intraocular, retroperitoneal, intraarticular or pericardial, or intramuscular with compartment syndrome, and/or *iii)* bleeding causing a fall in hemoglobin level of 20 g/L or more, or leading to transfusion of two or more units of whole blood or red cells (18). *Clinically relevant nonmajor bleeding* - clinically overt bleeding that did not satisfy the criteria for major bleeding and that led to hospital admission, physician-guided medical or surgical treatment, or a change in antithrombotic therapy (17).

**Pulmonary embolism -** confirmed by computed tomography-pulmonary angiogram in the presence of clinical symptoms (e.g. dyspnoea, chest pain, pre-syncope or syncope, and/or haemoptysis), electrocardiographic and laboratory abnormalities (e.g. elevated serum levels of D-dimer) (19).

**Time in therapeutic range (TTR)** – optional (if calculated as a part of the centre’s routine practice). The investigators were instructed to use the method of linear interpolation (Rosendaal method) when calculating TTR (20).

**Rerefences**

1. Hamm CW, Bassand J-P, Agewall S, Bax J, Boersma E, Bueno H, et al. ESC Guidelines for the management of acute coronary syndromes in patients presenting without persistent ST-segment elevation: The Task Force for the management of acute coronary syndromes (ACS) in patients presenting without persistent ST-segment elevatio. Eur Heart J. 2011;32:2999–3054.

2. Montalescot G, Sechtem U, Achenbach S, Andreotti F, Arden C, Budaj A, et al. 2013 ESC guidelines on the management of stable coronary artery disease: the Task Force on the management of stable coronary artery disease of the European Society of Cardiology. Eur Heart J. 2013;34:2949–3003.

3. Mancia G, Fagard R, Narkiewicz K, Redon J, Zanchetti A, Böhm M, et al. 2013 ESH/ESC guidelines for the management of arterial hypertension: the Task Force for the Management of Arterial Hypertension of the European Society of Hypertension (ESH) and of the European Society of Cardiology (ESC). Eur Heart J. 2013;34:2159–219.

4. McMurray JJ V, Adamopoulos S, Anker SD, Auricchio A, Böhm M, Dickstein K, et al. ESC Guidelines for the diagnosis and treatment of acute and chronic heart failure 2012: The Task Force for the Diagnosis and Treatment of Acute and Chronic Heart Failure 2012 of the European Society of Cardiology. Developed in collaboration with the Heart. Eur Heart J. 2012;33:1787–847. A

5. Vahanian A, Alfieri O, Andreotti F, Antunes MJ, Barón-Esquivias G, Baumgartner H, et al. Guidelines on the management of valvular heart disease (version 2012): the Joint Task Force on the Management of Valvular Heart Disease of the European Society of Cardiology (ESC) and the European Association for Cardio-Thoracic Surgery (EACTS). Eur J Cardiothorac Surg. 2012;42:S1–44.

6. Wynne J, Braunwal E. The Cardiomyopathies. In: Zipes DP, Libby P, Bonow RO, Braunwald E, editors. Braunwald's Heart Disease - A Textbook of Cardiovascular Medicine, 2^nd^ ed. Elservier-Saunders; 2005. p 1659-96.

7. Elliott PM, Anastasakis A, Borger MA, Borggrefe M, Cecchi F, Charron P, et al. 2014 ESC Guidelines on diagnosis and management of hypertrophic cardiomyopathy: The Task Force for the Diagnosis and Management of Hypertrophic Cardiomyopathy of the European Society of Cardiology (ESC). Eur Heart J. 2014;35:2733–79.

8. Web GD, Smallhorn JF, Therrien J, Redington AN. Congenital Heart Disease. In: Zipes DP, Libby P, Bonow RO, Braunwald E, editors. Braunwald's Heart Disease - A Textbook of Cardiovascular Medicine, 2^nd^ ed. Elservier-Saunders; 2005. p 1489-1552

9. Camm AJ, Kirchhof P, Lip GYH, Schotten U, Savelieva I, Ernst S, et al. Guidelines for the management of atrial fibrillation: the Task Force for the Management of Atrial Fibrillation of the European Society of Cardiology (ESC). Europace. 2010;12:1360–420.

10. Tendera M, Aboyans V, Bartelink M-L, Baumgartner I, Clément D, Collet J-P, et al. ESC Guidelines on the diagnosis and treatment of peripheral artery diseases: Document covering atherosclerotic disease of extracranial carotid and vertebral, mesenteric, renal, upper and lower extremity arteries: the Task Force on the Diagnosis and Treatm. Eur Heart J. 2011;32(22):2851–906.

11. Somers VK, White DP, Amin R, Abraham WT, Costa F, Culebras A, et al. Sleep apnea and cardiovascular disease: an American Heart Association/American College of Cardiology Foundation Scientific Statement from the American Heart Association Council for High Blood Pressure Research Professional Education Committee, Council on . J Am Coll Cardiol. 2008;52:686–717.

12. The Global Initiative for Chronic Obstructive Lung Disease (GOLD) - Global strategy for the diagnosis, management, and prevention of the chronic obstructive pulmonary disease, 2015 Update (http:// http://www.goldcopd.com accessed March 2015.

13. WHO. Haemoglobin concentrations for the diagnosis of anaemia and assessment of severity. Vitamin and Mineral Nutrition Information System. Geneva, World Health Organization, 2011 (WHO/NMH/NHD/MNM/11.1) (http://www.who.int/vmnis/indicators/haemoglobin. PDF, accessed March 2015).

14. Rydén L, Grant PJ, Anker SD, Berne C, Cosentino F, Danchin N, et al. ESC Guidelines on diabetes, pre-diabetes, and cardiovascular diseases developed in collaboration with the EASD: the Task Force on diabetes, pre-diabetes, and cardiovascular diseases of the European Society of Cardiology (ESC) and developed in collaboratio. Eur Heart J. 2013;34:3035–87.

15. Levey AS, Coresh J, Balk E, Kausz AT, Levin A, Steffes MW, et al. National Kidney Foundation practice guidelines for chronic kidney disease: evaluation, classification, and stratification. Ann Intern Med. 2003 Jul 15;139:137–47.

16. Heidelbaugh JJ, Bruderly M. Cirrhosis and chronic liver failure: part I. Diagnosis and evaluation. Am Fam Physician. 2006;74(5):756–62.

17. Lopes RD, Alexander JH, Al-Khatib SM, Ansell J, Diaz R, Easton JD, et al. Apixaban for reduction in stroke and other ThromboemboLic events in atrial fibrillation (ARISTOTLE) trial: design and rationale. Am Heart J. 2010;159:331–9.

18. Schulman S, Kearon C. Definition of major bleeding in clinical investigations of antihemostatic medicinal products in non-surgical patients. J Thromb Haemost. 2005;3:692–4.

19. Konstantinides S, Torbicki A, Agnelli G, Danchin N, Fitzmaurice D, Galie N, et al. 2014 ESC Guidelines on the diagnosis and management of acute pulmonary embolism: The Task Force for the Diagnosis and Management of Acute Pulmonary Embolism of the European Society of Cardiology (ESC) * Endorsed by the European Respiratory Society (ERS). Eur Heart J. 2014;35:3033–69.

20. Rosendaal FR, Cannegieter SC, van der Meer FJ, Briet E. A method to determine the optimal intensity of oral anticoagulant therapy. *Thrombosis and haemostasis*. 1993;69:236-239.

**APPENDIX 2**

**Supplemental tables**

Supplemental Table 1. Stroke and bleeding risk.

|  | **Total** | **Albania** | **B&H** | **Bulgaria** | **Croatia** | **Montenegro** | **Romania** | **Serbia** |
| --- | --- | --- | --- | --- | --- | --- | --- | --- |
|  | *n=2663* | *n=313* | *n=265* | *n=443* | *n=159* | *n=102* | *n=699* | *n=682* |
|  |  |  |  |  |  |  |  |  |
| *CHA_2_DS_2_-VASc score* | 3.48±1.78 | 3.37±1.79 | 3.66±1.78 | 3.71±1.76 | 3.21±1.89 | 2.54±1.61 | 3.81±1.64 | 3.20±1.76 |
| 0 | 90 (3.4) | 12 (3.8) | 7 (2.6) | 11 (2.5) | 9 (5.7) | 10 (9.8) | 12 (1.7) | 29 (4.3) |
| ‘truly low risk’* | 131 (4.9) | 21 (6.7) | 8 (3.0) | 14 (3.2) | 12 (7.5) | 12 (11.8) | 16 (2.3) | 48 (7.0) |
| 1 | 283 (10.6) | 38 (12.1) | 26 (9.8) | 36 (8.1) | 27 (17.0) | 17 (16.7) | 41 (5.9) | 98 (14.4) |
| ≥2 | 2290 (86.0) | 263 (84.0) | 232 (87.5) | 396 (89.4) | 123 (77.4) | 75 (73.5) | 646 (92.4) | 555 (81.4) |
|  |  |  |  |  |  |  |  |  |
| *CHADS_2_ score* | 2.15±1.29 | 2.05±1.34 | 2.30±1.34 | 2.35±1.29 | 1.96±1.34 | 1.49±1.07 | 2.37±1.23 | 1.90±1.24 |
| 0 | 201 (7.5) | 32 (10.2) | 13 (4.9) | 19 (4.3) | 22 (13.8) | 16 (15.7) | 32 (4.6) | 67 (9.8) |
| 1 | 715 (26.8) | 87 (27.8) | 73 (27.5) | 109 (24.6) | 42 (26.4) | 42 (41.2) | 131 (18.7) | 231 (33.9) |
| ≥2 | 1747 (65.6) | 194 (62.0) | 179 (67.5) | 315 (71.1) | 95 (59.7) | 44 (43.1) | 536 (76.7) | 384 (56.3) |
|  |  |  |  |  |  |  |  |  |
| *HASBLED score* | 1.97±1.23 | 1.87±1.28 | 1.91±1.18 | 1.92±1.12 | 1.77±1.17 | 1.87±1.22 | 2.25±1.26 | 1.86±1.23 |
| 0 | 275 (10.3) | 41 (13.3) | 27 (10.2) | 38 (8.6) | 15 (9.4) | 14 (13.7) | 49 (7.0) | 91 (13.3) |
| 1 | 728 (27.3) | 94 (30.0) | 76 (28.7) | 128 (28.9) | 63 (39.6) | 26 (25.5) | 147 (21.0) | 194 (28.4) |
| 2 | 838 (31.5) | 91 (29.1) | 88 (33.2) | 156 (35.2) | 42 (26.4) | 34 (33.3) | 227 (32.5) | 200 (29.3) |
| ≥3 | 822 (30.9) | 87 (27.8) | 74 (27.9) | 121 (27.3) | 39 (24.5) | 28 (27.5) | 276 (39.5) | 197 (28.9) |

*’Truly low risk’: patients with a CHA_2_DS_2_-VASc score of 0 and female patients with a CHA_2_DS_2_-VASc score of 1 by virtue of their gender.

**Supplemental Table 2. Full univariate analysis of factors influencing the use of antithrombotic therapies for stroke prevention in AF patients.**

| **Univariate analyses** | **OAC only** | | | **Single antiplatelet drug** | | | **DAPT** | | | **Dual or triple therapy** | | |
| --- | --- | --- | --- | --- | --- | --- | --- | --- | --- | --- | --- | --- |
|  | **OR** | **95%CI** | **P** | **OR** | **95%CI** | **P** | **OR** | **95%CI** | **P** | **OR** | **95%CI** | **P** |
| *Risk scores* |  |  |  |  |  |  |  |  |  |  |  |  |
| CHA_2_DS_2_-VASc (cont. variable) | 0.98 | 0.93-1.03 | 0.386 | 1.08 | 1.01-1.16 | 0.021 | 1.23 | 1.11-1.36 | <0.001 | 1.21 | 1.13-1.29 | <0.001 |
| CHA_2_DS_2_-VASc ≥2 | 1.17 | 0.91-1.50 | 0.196 | 1.41 | 0.98-2.03 | 0.062 | 2.23 | 1.12-4.45 | 0.022 | 2.26 | 1.43-3.58 | 0.001 |
| HASBLED (cont. variable) | 1.08 | 1.01-1.16 | 0.042 | 0.97 | 0.88-1.07 | 0.581 | 0.96 | 0.82-1.12 | 0.580 | 1.28 | 1.17-1.41 | <0.001 |
| HASBLED≥3 | 1.21 | 1.00-1.45 | 0.057 | 0.83 | 0.64-1.08 | 0.172 | 0.76 | 0.50-1.17 | 0.210 | 1.64 | 1.29-2.09 | <0.001 |
| *CHA_2_DS_2_-VASc score components* |  |  |  |  |  |  |  |  |  |  |  |  |
| Heart failure | 1.12 | 0.94-1.34 | 0.204 | 0.97 | 0.77-1.23 | 0.819 | 1.33 | 0.91-1.92 | 0.138 | 1.17 | 0.92-1.48 | 0.197 |
| Hypertension | 1.67 | 1.36-2.05 | <0.001 | 0.86 | 0.65-1.13 | 0.281 | 0.99 | 0.64-1.55 | 0.979 | 1.50 | 1.08-2.09 | 0.016 |
| Age 65-74 years | 1.14 | 0.94-1.38 | 0.171 | 0.83 | 0.64-1.07 | 0.146 | 1.09 | 0.74-1.61 | 0.659 | 1.13 | 0.88-1.45 | 0.329 |
| Age ≥75 years | 0.78 | 0.65-0.94 | 0.008 | 1.52 | 1.19-1.92 | 0.001 | 1.19 | 0.82-1.74 | 0.357 | 0.93 | 0.73-1.19 | 0.556 |
| Age≥80 years | 0.62 | 0.50-0.78 | <0.001 | 1.88 | 1.42-2.49 | <0.001 | 1.31 | 0.82-2.07 | 0.257 | 0.60 | 0.42-0.88 | 0.008 |
| Diabetes mellitus | 0.88 | 0.71-1.08 | 0.207 | 1.19 | 0.90-1.54 | 0.234 | 1.82 | 1.23-2.68 | 0.003 | 1.66 | 1.30-2.14 | <0.001 |
| Prior stroke/TIA | 1.00 | 0.77-1.31 | 0.986 | 1.19 | 0.84-1.67 | 0.330 | 1.19 | 0.70-2.02 | 0.251 | 1.14 | 0.81-1.59 | 0.463 |
| Prior stroke | 1.02 | 0.76-1.37 | 0.878 | 1.13 | 0.78-1.64 | 0.530 | 1.18 | 0.66-2.09 | 0.579 | 1.20 | 0.84-1.72 | 0.323 |
| Prior TIA | 0.79 | 0.48-1.28 | 0.332 | 1.78 | 1.01-3.13 | 0.047 | 0.78 | 0.24-2.52 | 0.681 | 0.86 | 0.42-1.74 | 0.672 |
| Vascular disease | 0.52 | 0.41-0.65 | <0.001 | 1.25 | 0.92-1.70 | 0.148 | 9.12 | 6.20-13.41 | <0.001 | 6.34 | 4.95-8.13 | <0.001 |
| Prior MI | 0.47 | 0.36-0.61 | <0.001 | 1.51 | 1.07-2.11 | 0.018 | 6.67 | 4.51-9.87 | <0.001 | 3.70 | 2.83-4.85 | <0.001 |
| PCI | 0.32 | 0.22-0.47 | <0.001 | 0.76 | 0.41-1.39 | 0.370 | 17.91 | 11.48-27.96 | <0.001 | 10.20 | 7.54-13.80 | <0.001 |
| CABG | 0.73 | 0.44-1.21 | 0.222 | 1.60 | 0.86-2.99 | 0.136 | 1.23 | 0.44-3.46 | 0.691 | 3.83 | 2.44-5.99 | <0.001 |
| PAD | 0.98 | 0.63-1.52 | 0.913 | 0.91 | 0.49-1.68 | 0.760 | 2.03 | 0.99-4.14 | 0.052 | 2.09 | 1.34-3.28 | 0.001 |
| Female gender | 1.05 | 0.88-1.26 | 0.571 | 1.13 | 0.89-1.43 | 0.321 | 0.74 | 0.51-1.08 | 0.122 | 0.72 | 0.56-0.91 | 0.007 |
| *Other cardiac conditions* |  |  |  |  |  |  |  |  |  |  |  |  |
| Coronary artery disease (any) | 0.49 | 0.41-0.60 | <0.001 | 1.60 | 1.24-2.06 | <0.001 | 14.39 | 9.03-22.92 | <0.001 | 5.99 | 4.65-7.73 | <0.001 |
| Stable coronary artery disease | 0.55 | 0.46-0.67 | <0.001 | 1.59 | 1.23-2.07 | <0.001 | 2.98 | 2.05-4.34 | <0.001 | 1.92 | 1.50-2.48 | <0.001 |
| Valvular heart disease | 1.34 | 1.11-1.61 | 0.003 | 0.77 | 0.60-1.00 | 0.048 | 0.65 | 0.43-0.99 | 0.044 | 0.82 | 0.63-1.05 | 0.110 |
| Mitral valve disease | 1.40 | 1.15-1.70 | 0.001 | 0.76 | 0.58-0.99 | 0.040 | 0.55 | 0.35-0.86 | 0.008 | 0.82 | 0.63-1.06 | 0.124 |
| Mitral valve regurgitation | 1.35 | 1.11-1.65 | 0.002 | 0.79 | 0.61-1.03 | 0.860 | 0.57 | 0.36-0.89 | 0.014 | 0.77 | 0.59-1.01 | 0.056 |
| Mitral valve stenosis | 6.32 | 1.50-26.56 | 0.012 | --- | --- | --- | 0.62 | 0.08-4.58 | 0.638 | 0.86 | 0.30-2.44 | 0.770 |
| Aortic valve disease | 0.92 | 0.70-1.21 | 0.540 | 1.60 | 1.16-2.21 | 0.005 | 0.76 | 0.40-1.42 | 0.385 | 0.56 | 0.38-0.91 | 0.016 |
| Dilated cardiomyopathy | 2.06 | 1.40-3.04 | <0.001 | 0.54 | 0.31-0.93 | 0.025 | 0.19 | 0.05-0.78 | 0.021 | 1.25 | 0.84-1.86 | 0.273 |
| Hypertrophic cardiomyopathy | 1.22 | 0.63-2.37 | 0.555 | 0.60 | 0.21-1.67 | 0.326 | 0.41 | 0.06-3.00 | 0.380 | 0.99 | 0.42-2.35 | 0.979 |
| Other heart disease | 1.18 | 0.84-1.66 | 0.352 | 0.48 | 0.27-0.85 | 0.012 | 0.75 | 0.34-1.63 | 0.468 | 1.22 | 0.88-1.86 | 0.358 |
| *Other conditions* |  |  |  |  |  |  |  |  |  |  |  |  |
| Body mass index | 1.06 | 1.04-1.08 | <0.001 | 0.96 | 0.93-0.99 | 0.003 | 0.97 | 0.92-1.01 | 0.131 | 1.03 | 1.01-1.06 | 0.020 |
| Chronic kidney disease | 1.06 | 0.83-1.36 | 0.651 | 0.89 | 0.63-1.24 | 0.480 | 1.22 | 0.75-1.99 | 0.419 | 1.25 | 0.92-1.70 | 0.156 |
| CKD on dialysis | 0.16 | 0.04-0.60 | 0.007 | 0.63 | 0.08-4.94 | 0.660 | 4.20 | 0.90-19.67 | 0.068 | 2.15 | 0.43-10.70 | 0.350 |
| COPD | 0.73 | 0.57-0.94 | 0.016 | 1.52 | 1.11-2.10 | 0.010 | 1.70 | 1.06-2.73 | 0.027 | 1.06 | 0.76-1.50 | 0.723 |
| Sleep apnoea | 1.26 | 0.63-2.50 | 0.522 | 1.68 | 0.80-3.56 | 0.168 | --- | --- | --- | 1.59 | 0.79-3.20 | 0.198 |
| Thyroid disease | 1.54 | 1.13-2.11 | 0.007 | 0.73 | 0.48-1.11 | 0.140 | 0.52 | 0.24-1.13 | 0.099 | 0.82 | 0.55-1.23 | 0.337 |
| Malignancy | 0.80 | 0.53-1.21 | 0.289 | 1.20 | 0.71-2.05 | 0.498 | 0.72 | 0.26-1.97 | 0.517 | 0.84 | 0.46-1.56 | 0.582 |
| Prior bleeding | 0.92 | 0.62-1.36 | 0.682 | 0.67 | 0.37-1.23 | 0.196 | 1.32 | 0.63-2.77 | 0.464 | 0.54 | 0.27-1.08 | 0.084 |
| *AF characteristics* |  |  |  |  |  |  |  |  |  |  |  |  |
| Known history of AF | 2.53 | 2.07-3.08 | <0.001 | 0.45 | 0.35-0.58 | <0.001 | 0.32 | 0.22-0.46 | <0.001 | 0.84 | 0.64-1.10 | 0.216 |
| Permanent AF | 1.80 | 1.49-2.17 | <0.001 | 0.56 | 0.44-0.73 | <0.001 | 0.54 | 0.36-0.82 | 0.003 | 0.93 | 0.73-1.18 | 0.531 |
| Paroxysmal AF | 0.38 | 0.32-0.46 | <0.001 | 2.28 | 1.80-2.89 | <0.001 | 3.12 | 2.13-4.56 | <0.001 | 1.01 | 0.79-1.30 | 0.915 |
| Asymptomatic AF | 1.22 | 0.98-1.52 | 0.082 | 0.76 | 0.56-1.04 | 0.085 | 0.52 | 0.30-0.91 | 0.021 | 1.07 | 0.80-1.42 | 0.648 |
| *Health-care related factors* |  |  |  |  |  |  |  |  |  |  |  |  |
| Centre in capital city | 2.02 | 1.68-2.43 | <0.001 | 0.40 | 0.31-0.52 | <0.001 | 0.77 | 0.52-1.12 | 0.166 | 1.30 | 1.03-1.65 | 0.027 |
| Hospital-based health centre | 1.04 | 0.76-1.43 | 0.806 | 0.74 | 0.50-1.10 | 0.136 | 0.68 | 0.38-1.21 | 0.186 | 3.06 | 1.60-5.85 | 0.001 |
| University centre | 2.09 | 1.62-2.69 | <0.001 | 0.50 | 0.37-0.69 | <0.001 | 0.93 | 0.53-1.63 | 0.796 | 0.98 | 0.68-1.40 | 0.903 |
| Cardiologist | 1.55 | 1.26-1.92 | <0.001 | 0.56 | 0.43-0.73 | <0.001 | 1.07 | 0.67-1.71 | 0.769 | 0.92 | 0.69-1.22 | 0.554 |

Heart failure defined by the CHA_2_DS_2_-VASc criteria; Dual therapy – OAC plus an antiplatelet drug; Triple therapy: OAC plus DAPT.

OAC: oral anticoagulant; DAPT: dual antiplatelet therapy; OR: Odds Ratio; CI: Confidence Interval; TIA: transient ischemic attack; MI: myocardial infarction; PCI: percutaneous coronary intervention; CABG: coronary artery bypass grafting; PAD: peripheral arterial disease; CKD: chronic kidney disease; COPD: chronic obstructive pulmonary disease; AF: atrial fibrillation.

**Supplemental Table 3. Full univariate analysis of factors influencing the use of OAC (alone or in any combination with other antithrombotic therapies) for stroke prevention in AF patients.**

|  | **OAC alone or in combination**  *Univariate analysis* | | |
| --- | --- | --- | --- |
| *Risk scores* | **OR** | **95%CI** | **P** |
| CHA_2_DS_2_-VASc (cont. variable) | 1.01 | 0.96-1.06 | 0.627 |
| CHA_2_DS_2_-VASc ≥2 | 1.31 | 1.03-1.66 | 0.027 |
| HASBLED (cont. variable) | 1.12 | 1.05-1.21 | 0.001 |
| HASBLED≥3 | 1.31 | 1.08-1.59 | 0.006 |
| *CHA_2_DS_2_-VASc score components* |  |  |  |
| Heart failure* | 1.15 | 0.97-1.37 | 0.109 |
| Hypertension | 1.76 | 1.44-2.15 | <0.001 |
| Age 65-74 years | 1.16 | 0.96-1.40 | 0.117 |
| Age ≥75 years | 0.78 | 0.66-0.94 | 0.007 |
| Age≥80 years | 0.59 | 0.47-0.74 | <0.001 |
| Diabetes mellitus | 0.97 | 0.80-1.19 | 0.974 |
| Prior stroke/TIA | 1.03 | 0.79-1.34 | 0.822 |
| Prior stroke | 1.06 | 0.80-1.41 | 0.684 |
| Prior TIA | 0.78 | 0.49-1.25 | 0.301 |
| Vascular disease | 0.86 | 0.70-1.06 | 0.150 |
| Prior MI | 0.72 | 0.56-0.91 | 0.006 |
| PCI | 0.93 | 0.69-1.27 | 0.650 |
| CABG | 1.09 | 0.68-1.75 | 0.706 |
| PAD | 1.16 | 0.76-1.77 | 0.502 |
| Female gender | 1.00 | 0.84-1.19 | 0.973 |
| *Other cardiac conditions* |  |  |  |
| Coronary artery disease (any) | 0.75 | 0.62-0.90 | 0.002 |
| Stable coronary artery disease | 0.73 | 0.59-0.89 | 0.002 |
| Valvular heart disease | 1.28 | 1.06-1.54 | 0.009 |
| Mitral valve disease | 1.34 | 1.11-1.62 | 0.003 |
| Mitral valve regurgitation | 1.29 | 1.06-1.56 | 0.010 |
| Mitral valve stenosis | 6.00 | 1.44-25.08 | 0.014 |
| Aortic valve disease | 0.86 | 0.66-1.12 | 0.262 |
| Dilated cardiomyopathy | 2.09 | 1.43-3.06 | <0.001 |
| Hypertrophic cardiomyopathy | 1.20 | 0.63-2.30 | 0.584 |
| Other heart disease | 1.20 | 0.86-1.67 | 0.294 |
| *Other conditions* |  |  |  |
| Body mass index | 1.06 | 1.04-1.09 | <0.001 |
| Chronic kidney disease | 1.10 | 0.86-1.40 | 0.442 |
| CKD on dialysis | 0.22 | 0.07-0.68 | 0.009 |
| COPD | 0.75 | 0.59-0.97 | 0.025 |
| Sleep apnoea | 1.38 | 0.71-2.69 | 0.350 |
| Thyroid disease | 1.48 | 1.08-2.01 | 0.013 |
| Malignancy | 0.78 | 0.53-1.17 | 0.230 |
| Prior bleeding | 0.85 | 0.58-1.25 | 0.849 |
| *AF characteristics* |  |  |  |
| Known history of AF | 2.32 | 1.92-2.81 | <0.001 |
| Permanent AF | 1.74 | 1.45-2.08 | <0.001 |
| Paroxysmal AF | 0.40 | 0.33-0.48 | <0.001 |
| Asymptomatic AF | 1.22 | 0.98-1.51 | 0.077 |
| *Health-care related factors* |  |  |  |
| Centre in capital city | 2.06 | 1.72-2.46 | <0.001 |
| Hospital-based health centre | 1.17 | 0.86-1.60 | 0.319 |
| University centre | 2.01 | 1.58-2.56 | <0.001 |
| Cardiologist | 1.50 | 1.22-1.84 | <0.001 |

*Heart failure defined by the CHA_2_DS_2_-VASc criteria; Dual therapy – OAC plus an antiplatelet drug; Triple therapy: OAC plus DAPT.

OAC: oral anticoagulant; DAPT: dual antiplatelet therapy; OR: Odds Ratio; CI: Confidence Interval; TIA: transient ischemic attack; MI: myocardial infarction; PCI: percutaneous coronary intervention; CABG: coronary artery bypass grafting; PAD: peripheral arterial disease; CKD: chronic kidney disease; COPD: chronic obstructive pulmonary disease; AF: atrial fibrillation.

**Supplemental Table 4. Univariate and multivariable analysis of factors influencing the use of OAC (alone or in any combination with other antithrombotic therapies) for stroke prevention in AF patients in whom AF was the main reason for enrolling visit or hospitalization (n=1329, 49.9%).**

| **OAC alone or in combination** | *Univariate analysis* | | | *Multivariable analysis* | | |
| --- | --- | --- | --- | --- | --- | --- |
| *Risk scores* | **OR** | **95%CI** | **P** | **OR** | **95%CI** | **P** |
| CHA_2_DS_2_-VASc (cont. variable) | 1.06 | 0.98-1.14 | 0.140 |  |  |  |
| CHA_2_DS_2_-VASc ≥2 | 1.50 | 1.13-1.99 | 0.005 |  |  |  |
| HASBLED (cont. variable) | 1.17 | 1.05-1.31 | 0.004 |  |  |  |
| HASBLED≥3 | 1.40 | 1.03-1.91 | 0.031 |  |  |  |
| *CHA_2_DS_2_-VASc score components* |  |  |  |  |  |  |
| Heart failure* | 1.04 | 0.79-1.36 | 0.802 |  |  |  |
| Hypertension | 1.83 | 1.38-2.43 | <0.001 | 1.97 | 1.42-2.73 | <0.001 |
| Age 65-74 years | 1.42 | 1.08-1.88 | 0.012 | 1.46 | 1.06-2.02 | 0.023 |
| Age ≥75 years | 0.71 | 0.54-0.93 | 0.013 |  |  |  |
| Age≥80 years | 0.64 | 0.44-0.92 | 0.015 |  |  |  |
| Diabetes mellitus | 1.03 | 0.75-1.43 | 0.838 |  |  |  |
| Prior stroke/TIA | 1.29 | 0.85-1.95 | 0.237 |  |  |  |
| Prior stroke | 1.39 | 0.87-2.24 | 0.171 |  |  |  |
| Prior TIA | 0.93 | 0.48-1.81 | 0.827 |  |  |  |
| Vascular disease | 1.34 | 0.90-2.00 | 0.148 |  |  |  |
| Prior MI | 0.79 | 0.49-1.28 | 0.339 |  |  |  |
| PCI | 1.79 | 0.90-3.55 | 0.098 |  |  |  |
| CABG | 0.87 | 0.40-1.90 | 0.735 |  |  |  |
| PAD | 1.93 | 0.90-4.13 | 0.090 |  |  |  |
| Female gender | 1.06 | 0.83-1.34 | 0.627 |  |  |  |
| *Other cardiac conditions* |  |  |  |  |  |  |
| Coronary artery disease (any) | 0.94 | 0.69-1.30 | 0.720 |  |  |  |
| Stable coronary artery disease | 0.78 | 0.55-1.10 | 0.156 |  |  |  |
| Valvular heart disease | 1.30 | 0.97-1.74 | 0.084 |  |  |  |
| Mitral valve disease | 1.44 | 1.06-1.97 | 0.021 |  |  |  |
| Mitral valve regurgitation | 1.40 | 1.02-1.92 | 0.036 |  |  |  |
| Mitral valve stenosis | 4.66 | 0.61-35.55 | 0.138 |  |  |  |
| Aortic valve disease | 0.75 | 0.48-1.16 | 0.192 |  |  |  |
| Dilated cardiomyopathy | 2.20 | 1.03-4.68 | 0.041 |  |  |  |
| Hypertrophic cardiomyopathy | 1.54 | 0.44-5.40 | 0.500 |  |  |  |
| Other heart disease | 1.05 | 0.64-1.73 | 0.846 |  |  |  |
| *Other conditions* |  |  |  |  |  |  |
| Body mass index | 1.06 | 1.03-1.10 | 0.001 | 1.05 | 1.01-1.09 | 0.009 |
| Chronic kidney disease | 0.92 | 0.60-1.41 | 0.689 |  |  |  |
| CKD on dialysis | 0.13 | 0.03-0.67 | 0.015 |  |  |  |
| COPD | 0.49 | 0.33-0.73 | <0.001 | 0.50 | 0.30-0.82 | 0.006 |
| Sleep apnoea | 0.43 | 0.15-1.26 | 0.125 |  |  |  |
| Thyroid disease | 1.73 | 1.13-2.65 | 0.012 | 1.83 | 1.11-3.02 | 0.017 |
| Malignancy | 0.56 | 0.32-0.97 | 0.040 | 0.38 | 0.20-0.72 | 0.003 |
| Prior bleeding | 0.99 | 0.54-1.79 | 0.964 |  |  |  |
| *AF characteristics* |  |  |  |  |  |  |
| Known history of AF | 2.42 | 1.86-3.12 | <0.001 |  |  |  |
| Permanent AF | 1.57 | 1.16-2.12 | 0.004 |  |  |  |
| Paroxysmal AF | 0.41 | 0.31-0.52 | <0.001 | 0.34 | 0.25-0.46 | <0.001 |
| Asymptomatic AF | 1.37 | 1.03-1.84 | 0.034 |  |  |  |
| *Health-care related factors* |  |  |  |  |  |  |
| Centre in capital city | 2.29 | 1.76-2.98 | <0.001 | 2.15 | 1.55-2.98 | <0.001 |
| Hospital-based health centre | 1.44 | 0.99-2.08 | 0.055 |  |  |  |
| University centre | 2.47 | 1.73-3.53 | <0.001 | 1.68 | 1.11-2.53 | 0.014 |
| Cardiologist | 1.42 | 1.07-1.82 | 0.015 |  |  |  |

*Heart failure defined by the CHA_2_DS_2_-VASc criteria; OAC: oral anticoagulant; DAPT: dual antiplatelet therapy; OR: Odds Ratio; CI: Confidence Interval; TIA: transient ischemic attack; MI: myocardial infarction; PCI: percutaneous coronary intervention; CABG: coronary artery bypass grafting; PAD: peripheral arterial disease; CKD: chronic kidney disease; COPD: chronic obstructive pulmonary disease; AF: atrial fibrillation.

**Supplemental Table 5. Univariate and multivariable analysis of factors influencing the use of OAC (alone or in any combination with other antithrombotic therapies) for stroke prevention in AF patients, excluding those with an acute coronary syndrome (n=206, 7.7%).**

| **OAC alone or in combination** | *Univariate analysis* | | | *Multivariable analysis* | | |
| --- | --- | --- | --- | --- | --- | --- |
| *Risk scores* | **OR** | **95%CI** | **P** | **OR** | **95%CI** | **P** |
| CHA_2_DS_2_-VASc (cont. variable) | 1.03 | 0.98-1.09 | 0.255 |  |  |  |
| CHA_2_DS_2_-VASc ≥2 | 1.38 | 1.08-1.76 | 0.010 |  |  |  |
| HASBLED (cont. variable) | 1.12 | 1.03-1.20 | 0.005 |  |  |  |
| HASBLED≥3 | 1.30 | 1.06-1.59 | 0.013 | 1.32 | 1.04-1.67 | 0.024 |
| *CHA_2_DS_2_-VASc score components* |  |  |  |  |  |  |
| Heart failure* | 1.24 | 1.04-1.50 | 0.019 |  |  |  |
| Hypertension | 1.76 | 1.42-2.17 | <0.001 | 1.80 | 1.42-2.28 | <0.001 |
| Age 65-74 years | 1.16 | 0.95-1.41 | 0.139 |  |  |  |
| Age ≥75 years | 0.78 | 0.64-0.94 | 0.008 |  |  |  |
| Age≥80 years | 0.58 | 0.46-0.74 | <0.001 | 0.51 | 0.38-0.68 | <0.001 |
| Diabetes mellitus | 1.03 | 0.83-1.27 | 0.822 |  |  |  |
| Prior stroke/TIA | 1.08 | 0.82-1.42 | 0.602 |  |  |  |
| Prior stroke | 1.12 | 0.82-1.51 | 0.481 |  |  |  |
| Prior TIA | 0.76 | 0.46-1.23 | 0.261 |  |  |  |
| Vascular disease | 1.03 | 0.81-1.31 | 0.788 |  |  |  |
| Prior MI | 0.88 | 0.67-1.17 | 0.329 |  |  |  |
| PCI | 1.13 | 0.77-1.67 | 0.533 |  |  |  |
| CABG | 0.95 | 0.58-1.56 | 0.847 |  |  |  |
| PAD | 1.08 | 0.69-1.68 | 0.744 |  |  |  |
| Female gender | 0.97 | 0.81-1.16 | 0.735 |  |  |  |
| *Other cardiac conditions* |  |  |  |  |  |  |
| Coronary artery disease (any) | 0.87 | 0.71-1.07 | 0.186 |  |  |  |
| Stable coronary artery disease | 0.81 | 0.65-1.02 | 0.812 |  |  |  |
| Valvular heart disease | 1.27 | 1.05-1.55 | 0.015 |  |  |  |
| Mitral valve disease | 1.33 | 1.09-1.63 | 0.006 |  |  |  |
| Mitral valve regurgitation | 1.27 | 1.04-1.56 | 0.020 |  |  |  |
| Mitral valve stenosis | 5.58 | 1.34-23.32 | 0.018 | 6.03 | 1.39-26.06 | 0.016 |
| Aortic valve disease | 0.88 | 0.66-1.16 | 0.357 |  |  |  |
| Dilated cardiomyopathy | 1.94 | 1.32-2.87 | 0.001 | 1.78 | 1.16-2.72 | 0.008 |
| Hypertrophic cardiomyopathy | 1.09 | 0.56-2.09 | 0.808 |  |  |  |
| Other heart disease | 1.13 | 0.80-1.60 | 0.494 |  |  |  |
| *Other conditions* |  |  |  |  |  |  |
| Body mass index | 1.06 | 1.04-1.09 | <0.001 | 1.06 | 1.03-1.08 | <0.001 |
| Chronic kidney disease | 1.09 | 0.85-1.42 | 0.494 |  |  |  |
| CKD on dialysis | 0.21 | 0.07-0.63 | 0.006 |  |  |  |
| COPD | 0.77 | 0.59-0.99 | 0.045 |  |  |  |
| Sleep apnoea | 1.18 | 0.60-2.33 | 0.624 |  |  |  |
| Thyroid disease | 1.39 | 1.01-1.90 | 0.045 | 1.52 | 1.05-2.21 | 0.026 |
| Malignancy | 0.73 | 0.48-1.10 | 0.130 |  |  |  |
| Prior bleeding | 0.79 | 0.53-1.16 | 0.231 |  |  |  |
| *AF characteristics* |  |  |  |  |  |  |
| Known history of AF | 2.14 | 1.74-2.63 | <0.001 |  |  |  |
| Permanent AF | 1.66 | 1.37-2.06 | <0.001 |  |  |  |
| Paroxysmal AF | 0.43 | 0.35-0.51 | <0.001 | 0.38 | 0.31-0.47 | <0.001 |
| Asymptomatic AF | 1.20 | 0.95-1.50 | 0.120 |  |  |  |
| *Health-care related factors* |  |  |  |  |  |  |
| Centre in capital city | 2.11 | 1.74-2.56 | <0.001 | 2.05 | 1.62-2.58 | <0.001 |
| Hospital-based health centre | 1.27 | 0.93-1.74 | 0.132 |  |  |  |
| University centre | 2.05 | 1.59-2.64 | <0.001 | 1.60 | 1.20-2.14 | 0.001 |
| Cardiologist | 1.50 | 1.21-1.86 | <0.001 |  |  |  |

*Heart failure defined by the CHA_2_DS_2_-VASc criteria; OAC: oral anticoagulant; DAPT: dual antiplatelet therapy; OR: Odds Ratio; CI: Confidence Interval; TIA: transient ischemic attack; MI: myocardial infarction; PCI: percutaneous coronary intervention; CABG: coronary artery bypass grafting; PAD: peripheral arterial disease; CKD: chronic kidney disease; COPD: chronic obstructive pulmonary disease; AF: atrial fibrillation.
